# Mitochondrion of the *Trypanosoma brucei* long slender bloodstream form is capable of ATP production by substrate-level phosphorylation

**DOI:** 10.1371/journal.ppat.1011699

**Published:** 2023-10-11

**Authors:** Gergana Taleva, Michaela Husová, Brian Panicucci, Carolina Hierro-Yap, Erika Pineda, Marc Biran, Martin Moos, Petr Šimek, Falk Butter, Frédéric Bringaud, Alena Zíková

**Affiliations:** 1 Institute of Parasitology, Biology Centre CAS, Ceske Budejovice, Czech republic; 2 Faculty of Science, University of South Bohemia, Ceske Budejovice, Czech republic; 3 Univ. Bordeaux, CNRS, Laboratoire de Microbiologie Fondamentale et Pathogénicité (MFP), Université de Bordeaux, Bordeaux, France; 4 Univ. Bordeaux, CNRS, Centre de Résonance Magnétique des Systèmes Biologiques (CRMSB), Bordeaux, France; 5 Institute of Entomology, Biology Centre CAS, Ceske Budejovice, Czech republic; 6 Institute of Molecular Biology (IMB), Mainz, Germany; 7 Institute of Molecular Virology and Cell Biology, Friedrich-Loeffler-Institute, Greifswald, Germany; University at Buffalo School of Medicine and Biomedical Sciences, UNITED STATES

## Abstract

The long slender bloodstream form *Trypanosoma brucei* maintains its essential mitochondrial membrane potential (ΔΨm) through the proton-pumping activity of the F_o_F_1_-ATP synthase operating in the reverse mode. The ATP that drives this hydrolytic reaction has long been thought to be generated by glycolysis and imported from the cytosol via an ATP/ADP carrier (AAC). Indeed, we demonstrate that AAC is the only carrier that can import ATP into the mitochondrial matrix to power the hydrolytic activity of the F_o_F_1_-ATP synthase. However, contrary to expectations, the deletion of AAC has no effect on parasite growth, virulence or levels of ΔΨ_m_. This suggests that ATP is produced by substrate-level phosphorylation pathways in the mitochondrion. Therefore, we knocked out the succinyl-CoA synthetase (SCS) gene, a key mitochondrial enzyme that produces ATP through substrate-level phosphorylation in this parasite. Its absence resulted in changes to the metabolic landscape of the parasite, lowered virulence, and reduced mitochondrial ATP content. Strikingly, these SCS mutant parasites become more dependent on AAC as demonstrated by a 25-fold increase in their sensitivity to the AAC inhibitor, carboxyatractyloside. Since the parasites were able to adapt to the loss of SCS in culture, we also analyzed the more immediate phenotypes that manifest when SCS expression is rapidly suppressed by RNAi. Importantly, when performed under nutrient-limited conditions mimicking various host environments, SCS depletion strongly affected parasite growth and levels of ΔΨ_m_. In totality, the data establish that the long slender bloodstream form mitochondrion is capable of generating ATP via substrate-level phosphorylation pathways.

## Author summary

In the bloodstream of a mammalian host, proliferating *Trypanosoma brucei* parasites take up glucose and generate most of their ATP by glycolysis. This is atypical for an aerobic eukaryotic cell, which usually employes mitochondrial oxidative phosphorylation to generate ATP. In this unique case, the mitochondrion of the *T*. *brucei* bloodstream form has lost its function as the powerhouse of the cell, and the organelle of the parasite has been considered to be only an ATP consumer. However, we have shown that this is not entirely correct and that the parasite mitochondrion can produce ATP itself by phosphorylation at the substrate level. We have mapped the possible metabolic pathways and identified a key enzyme responsible for this activity: succinyl-coenzyme A synthetase. The importance of this enzyme for parasite viability depends on culture media that mimic different mammalian host environments. Our study offers a revolutionary new insight into bloodstream form mitochondrial metabolism and provides a deeper understanding of the parasite mitochondrion, which is the target of commonly used cationic drugs to treat African Animal trypanosomiasis.

## Introduction

The unicellular parasite, *Trypanosoma brucei*, dramatically rewires its metabolism as it undergoes a complex digenetic life cycle through the tsetse insect vector and various mammalian hosts [1]. This advantageous adaptation is in response to the varied nutrients encountered as the extracellular parasite first traverses from the tsetse midgut to the salivary glands before invading the bloodstream and interstitial spaces of distinct mammalian organs and tissues [2]. In terms of energy metabolism, the insect forms of the parasite mainly consume amino acids (e.g. proline, threonine), which are oxidized in its single mitochondrion to succinate, acetate and alanine [3–5]. This generates ATP by both the oxidative and substrate-level phosphorylation pathways [6–9]. On the contrary, the dividing long slender bloodstream form (abbreviated hereafter as BSF) resides in the glucose-rich environment of the mammalian bloodstream and synthesizes the majority of their cellular ATP in the cytosol through robust glycolysis [10]. This bioenergetic switch is essential because the proton-pumping electron transport chain (ETC) complexes III and IV are absent [11] and complex I does not contribute to the mitochondrial proton motive force in this life cycle stage [12]. Without functional complexes III and IV, the BSF expresses the Trypanosoma alternative oxidase (TAO or AOX) [13]. While this enzyme transfers electrons from ubiquinol to oxygen, it does not generate a proton motive force. Therefore, ATP cannot be produced by oxidative phosphorylation via the F_o_F_1_-ATP synthase. Consequently, the BSF is a rare example of an aerobic organism that does not employ its mitochondrion as the powerhouse of the cell [14].

Lacking the enzymes that normally participate in generating the proton motive force, the conditions dictate that the F_o_F_1_-ATP synthase operates in the reverse mode. By hydrolyzing ATP and utilizing the released energy to pump protons across the inner mitochondrial membrane, this elegant enzyme maintains the BSF mitochondrial membrane potential (ΔΨ_m_) [15,16]. The reverse activity of this multi-subunit protein complex is well known in the aerobic eukaryote world, but it is usually employed only for a short period of time to overcome sudden changes in the environment (e.g. hypoxia or anoxia conditions) that result in impaired respiration and mitochondrial membrane depolarization [17]. Under these conditions, the F_o_F_1_-ATP synthase first reverses its rotation and hydrolyzes the ATP supplied by mitochondrial substrate-level phosphorylation, a rescue mechanism that protects against cytosolic ATP depletion [18,19]. However, if the intramitochondrial ATP/ADP ratio decreases and the ΔΨ_m_ is reduced even more, the ATP/ADP carrier (AAC) also reverses. These circumstances can deplete cellular ATP as cytosolic ATP is imported into the mitochondrion to supply the substrate needed for the F_o_F_1_-ATPase activity. This scenario can rapidly lead to cell death if the F_o_F_1_-ATPase activity is not constrained by the inhibitory peptide IF1 [20,21].

Uniquely, *T*. *brucei* is capable of exploiting the reverse mode of the enzyme for long periods of time. The hydrolytic activity of F_o_F_1_-ATP synthase appears to be the only entity that generates the ΔΨ_m_ in BSF, as RNAi silencing of its subunits causes a decrease in ΔΨ_m_ within 24 hours [16,22,23]. Furthermore, inhibition of ATP hydrolysis by the forced expression of the *T*. *brucei* homolog of the inhibitory peptide IF1 (TbIF1) decreases the ΔΨ_m_ below the BSF viability threshold within 12 hours [24]. Thus, the BSF F_o_F_1_-ATP synthase is not an ATP-producing enzyme but an ATP-consuming enzyme. Therefore, we were intrigued to decipher which metabolic pathways supply this molecular nanomachine with ATP. There are at least two possibilities: either ATP is taken from the cytosol and imported into the mitochondrial matrix by an ATP/ADP carrier [25,26] or the mitochondrion produces the ATP itself through substrate-level phosphorylation pathways. Because the mitochondrion of the BSF is metabolically poor when compared with the insect forms [27], it was proposed that the organelle does not participate in ATP production and that the glycolytically produced ATP is imported from the cytosol. However, there has been no direct experimental evidence for this assumption.

**Fig 1:**
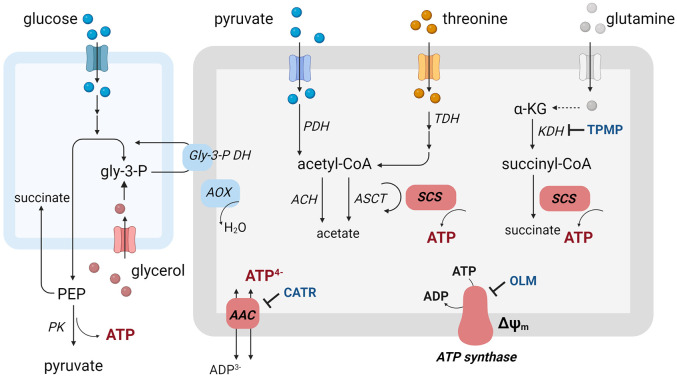
Schematic and simplified representation of possible metabolic pathways related to cytosolic and mitochondrial ATP production. Enzymes are: ACH, acetyl-CoA thioesterase; AOX, alternative oxidase; ASCT, acetate:succinate CoA-transferase, Gly-3-P DH, glycerol-3-phosphate dehydrogenase; KDH, α-ketoglutarate dehydrogenase; PK, pyruvate kinase; PDH, pyruvate dehydrogenase complex; SCS, succinyl-CoA synthetase; TDH, threonine dehydrogenase. Abbreviations: α-KG, α-ketoglutarate; ΔΨm, mitochondrial membrane potential; AAC, ATP/ADP carrier; CATR, carboxyatractyloside; OLM, oligomycin; TPMP, methyltriphenylphosphonium.

Remarkably, the BSF parasites exhibit ~40-fold lower sensitivity to AAC inhibitors than dyskinetoplastic trypanosomes, which lack their mitochondrial genome and thus the proton-pumping F_o_F_1_-ATPase activity [28]. Even though these dyskinetoplastic trypanosomes fully rely on the electrogenic exchange of ATP^4-^/ADP^3-^ to generate ΔΨ_m_ [29], the striking difference in sensitivity to AAC inhibitors raises questions about the role of AAC for BSF mitochondria. Moreover, new metabolomic and proteomic data suggest that the metabolic potential of the BSF parasite mitochondrion may be greater than originally thought and may potentially contribute to intramitochondrial ATP production [30–32]. For example, it was recently established that a portion of glucose-derived pyruvate and threonine are further metabolized to acetate, an essential precursor for *de novo* fatty acid synthesis [33]. Glucose-derived pyruvate and threonine are metabolized by pyruvate- and threonine dehydrogenases (PDH and TDH), respectively, leading to the formation of acetyl-Coenzyme A (acetyl-CoA). This energy-rich compound is rapidly converted to acetate by two redundant pathways. The first employs acetyl-CoA thioesterase (ACH). The second utilizes acetate:succinate-CoA transferase (ASCT), which is coupled to succinyl-CoA synthetase (SCS) activity to simultaneously produce mitochondrial ATP [34] (Fig 1). Isotope-labeled metabolomic data have also shown production of succinate that is not derived from glucose, suggesting that other carbon sources can be metabolized, such as amino acids [32]. Interestingly, the BSF consumes significant levels of glutamine from the medium [31]. Glutamine-derived α-ketoglutarate can be converted by α-ketoglutarate dehydrogenase (KDH) to succinyl-CoA, which is the substrate for ATP-producing SCS. Moreover, α-ketoglutarate can be produced by amino acid transaminases. As a precedence, the transitional short stumpy bloodstream form, cell-cycle arrested parasites primed for the transmission to the insect host, maintains high levels of intracellular ATP in the presence α-ketoglutarate possibly by the substrate phosphorylation [35]. Thus, it is plausible that the mitochondrion of the proliferative long slender BSF may be also capable of intramitochondrial ATP production (Fig 1).

African trypanosomes (*T*. *brucei spp*, *T*. *congolense*, *T*. *vivax*) are parasites of great medical and veterinary importance. While Human African Trypanosomiasis (HAT) is expected to be eliminated as a public health problem by 2025 [36], Animal African Trypanosomiasis (AAT) represents an enormous economic burden. While AAT is commonly treated with cationic drugs (e.g. isometamidium and diminazene) that are sequestered within the mitochondrion [37,38], resistance to some of these drugs is linked to parasites with a reduced ΔΨ_m_ [39–41]. Therefore, it is critical to understand the molecular mechanisms responsible for the maintenance of the ΔΨ_m_ in the infectious forms of trypanosomes. To determine which molecular entities supply ATP to the reversed F_o_F_1_-ATP synthase, we generated two different null mutant cell lines in which AAC or SCS was eliminated. Evaluating how the absence of these gene products affects the viability, bioenergetics and the mitochondrial metabolism of *T*. *brucei* BSF parasites revealed that the BSF mitochondrion is capable of ATP production.

## Results

### ATP/ADP carrier is dispensable in BSF *T*. *brucei in vitro* and *in vivo*

**Fig 2:**
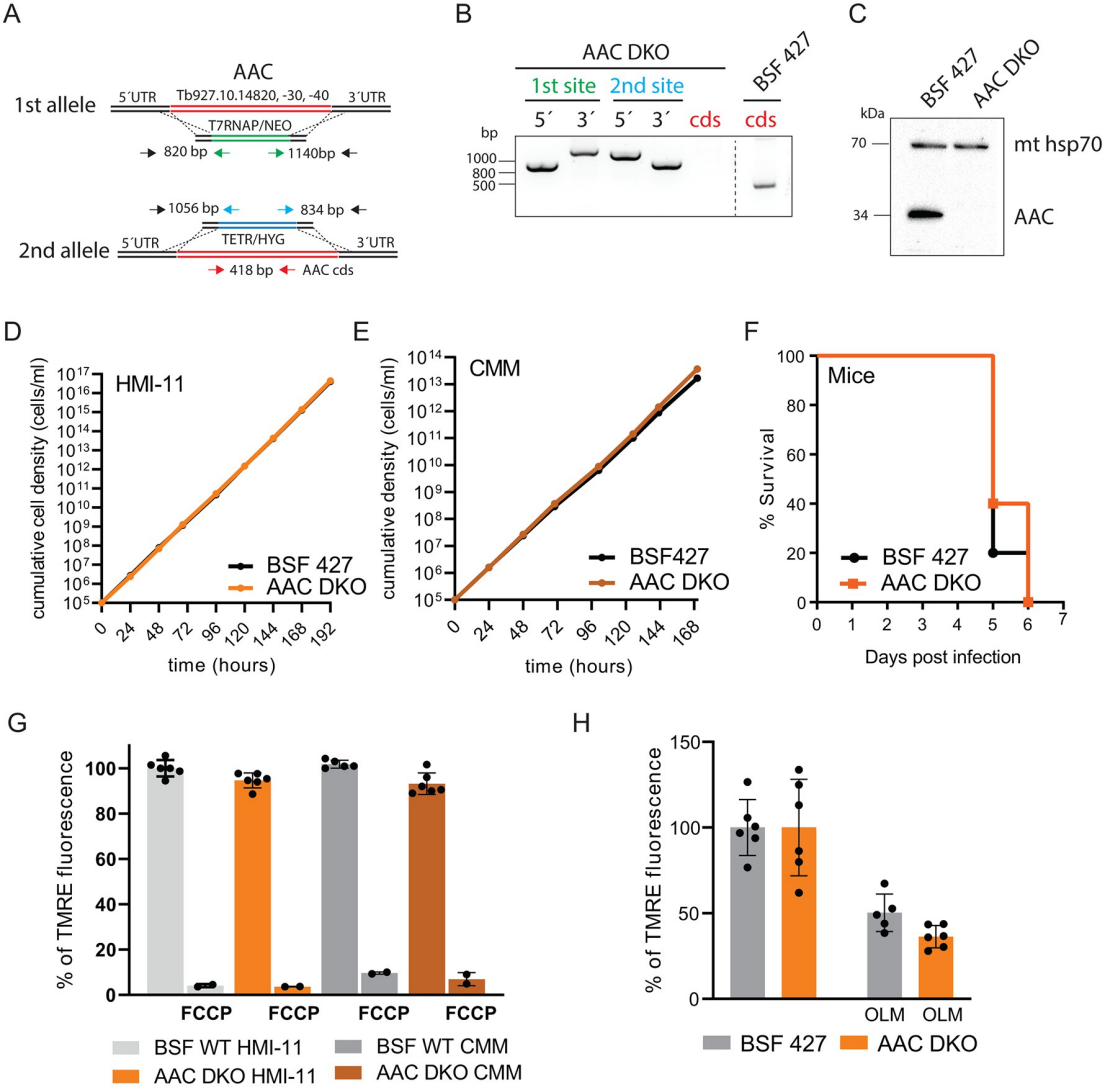
The ATP/ADP carrier is dispensable for BSF *T*. *brucei* viability and for maintaining the ΔΨm. (A) The strategy to generate AAC DKO involved replacement of both alleles with T7 RNA polymerase and tetracycline repressor linked to genes conferring neomycin and hygromycin resistance, respectively. (B) PCR verification for the elimination of all AAC alleles in AAC DKO cell line. The primers used are color-coded in (A). (C) Immunoblot analysis of AAC DKO cells using specific anti-AAC antibody. Immunodetection of mitochondrial hsp 70 served as a loading control. (D) Growth of AAC DKO cells compared to wild-type BSF 427 in HMI-11 measured for 8 days. (E) Growth of AAC DKO cells compared to wild-type BSF 427 in CMM medium measured for 7 days. (F) The survival rate of 5 female BALB/c mice which were intraperitoneally infected with AAC DKO and wild-type BSF 427 parasites. The infected mice were monitored for 6 days. (G) Flow cytometry analysis of TMRE-stained AAC DKO and BSF 427 cells grown in HMI-11 or CMM medium to measure ΔΨm. The addition of FCCP served as a control for ΔΨ_m_ depolarization (+FCCP). (means ± s.d., n = 6). (H) Flow cytometry analysis of TMRE-stained AAC DKO and BSF 427 cells grown in HMI-11 medium and treated with 250 ng/ml of oligomycin (+OLM) for 24 hours before the analysis. (means ± s.d., n = 6).

The *T*. *brucei* ATP/ADP carrier (AAC, originally named MCP5 [25]) is represented by three identical and consecutive genes (Tb927.10.14820, -14830, -14840) in the parasite genome. To determine if the replicative long slender BSF viability depends on the presence of AAC, we removed all three genes by homologous recombination, resulting in an AAC double knock-out mutant (AAC DKO) (Fig 2A). We verified the correct genomic integration of the two cassettes containing antibiotic resistance genes by PCR (Fig 2B) and by Western blot using a specific polyclonal antibody raised against recombinant *T*. *brucei* AAC [26] (Fig 2C). The AAC DKO mutants showed no significant growth effect when grown in the commonly used HMI-11 medium containing a high concentration of glucose (25 mM) (Fig 2D). The same lack of growth phenotype was observed in the simplified Creek minimal medium (CMM), which, with its 10 mM glucose, represents better, although still well above, the extracellular glucose concentration in the mammalian host [42] (Fig 2E). Next, we examined the virulence of AAC mutant parasites by infecting two groups of BALB/c mice with either the parental (BSF 427) or AAC DKO *T*. *brucei*. The parasitemia levels and the rate of survival were monitored over several days. Neither group of infected mice survived beyond day 6, indicating that the AAC DKO mutants are fully virulent in the mouse model (Fig 2F) and thus AAC is dispensable for BSF parasite viability.

Since the *T*. *brucei* BSF mitochondrion has long been assumed to be strictly an ATP consuming organelle, it was predicted that AAC functions in the reverse mode to supply the mitochondrial matrix with cytosolic ATP. Once in the mitochondrion, the ATP is hydrolyzed by F_o_F_1_-ATP synthase to maintain the essential ΔΨ_m_. To examine this assumption, we investigated whether the absence of AAC affects ΔΨ_m_ in living *T*. *brucei* parasites. For this purpose, we used the fluorescent lipophilic dye, tetramethylrhodamine ethyl ester (TMRE), in a non-quenching mode to analyze the stained parasites by flow cytometry. We detected no difference in the fluorescence intensity averaged over the entire cell population of BSF 427 and AAC DKO cells grown in either HMI-11 or CMM media. This demonstrates that AAC DKO parasites maintain their ΔΨ_m_ at the same level as BSF 427. Treatment with FCCP, a protonophore, induced membrane depolarization as expected (Fig 2G). To determine if the AAC DKO mutants still maintain their ΔΨ_m_ by the reverse activity of F_o_F_1_-ATP synthase, the BSF 427 and AAC DKO cells were incubated for 24 hours with a sublethal concentration of the F_o_F_1_-ATP synthase inhibitor, oligomycin (250 ng/ml, ~0.5 of the EC_50_ for BSF 427 [22,28]). While this treatment did not affect the doubling time of BSF 427 or AAC DKO parasites (BSF 427: 6 ± 0.2 hours, AAC DKO: 6.3 ± 0.3), it did result in a similar reduction of the ΔΨ_m_ in BSF 427 and AAC DKO mutants, with values reaching 50±11% and 64±7%, respectively (Fig 2H). Moreover, Alamar Blue assays revealed that the AAC DKO mutant remains sensitive to oligomycin, with EC_50_ values even lower than BSF 427 (BSF 427 EC_50_: 0.489 μg/ml, AAC DKO EC_50_: 0.155 μg/ml). These results indicate that the AAC DKO cells still rely on the reverse F_o_F_1_-ATP synthase activity to maintain their ΔΨ_m_.

### AAC DKO is unable to import ATP into the mitochondrial matrix

**Fig 3:**
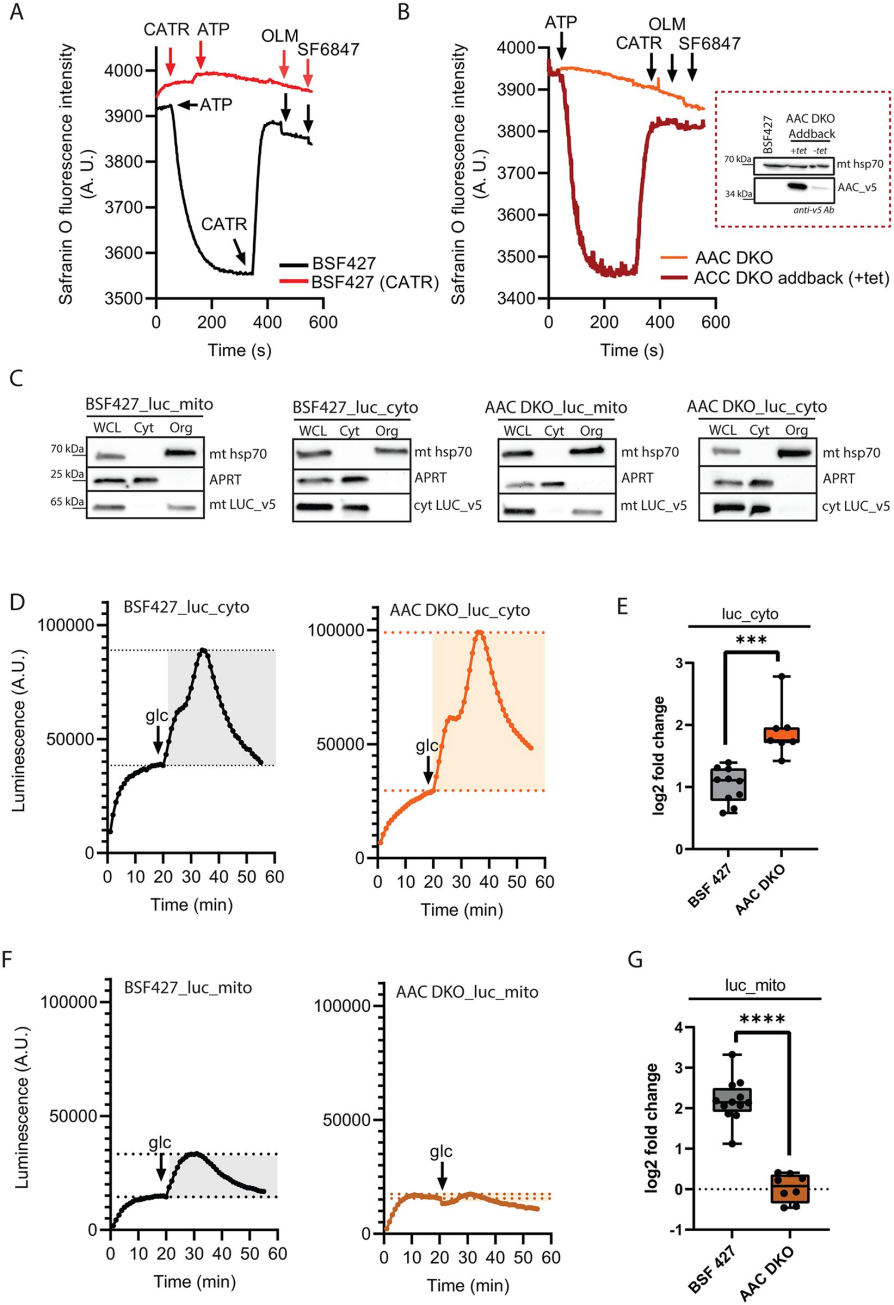
In the absence of AAC, the cells are unable to import cytosolic ATP to the mitochondrial matrix. (A) Mitochondrial membrane polarization detected using Safranine O dye in digitonin-permeabilized BSF 427 cells in the presence of ATP. Carboxyatractyloside (CATR), the AAC inhibitor was added before the ATP (red line) as a control for no membrane polarization due to the inability to import ATP into the mitochondrial matrix. Oligomycin (OLM) was added after the CATR to induce depolarization. SF6847, an uncoupler, was added to test any further depolarization. ATP, CATR, OLM and SF 6847 were added where indicated. (B) Mitochondrial membrane polarization detected using Safranine O dye in digitonin-permeabilized AAC DKO and AAC DKO Addback cells in the presence of ATP. CATR, OLM and SF 6847 were added where indicated. The inset shows western blot analysis of BSF 427, AAC DKO Addback cells grown in the presence or absence of tetracycline, probed with anti-v5 monoclonal antibody, that recognizes the v5 epitope attached to the 3´end of the *AAC* gene, and anti-mt Hsp70 antibody as a loading control. (C) Subcellular localization of v5-tagged luciferase without (luc_cyto) or with mitochondrial localization signal (luc_mito) endogenously expressed in BSF 427 and AAC DKO cells was determined in whole cell lysates and in the corresponding cytosolic and organellar fractions separated by digitonin extraction. Purified fractions were analyzed by Western blotting with the following antibodies: anti-v5, anti-mt Hsp70 (mitochondrial marker), and anti-adenosine phosphoribosyltransferase (APRT) (cytosolic marker). The relevant sizes of the protein marker are indicated on the left. (D) Representative data of basal (first peak) and glucose-induced (second peak) levels of bioluminescence detected by a plate reader in the cytosol of BSF 427_luc_cyto (left panel) and AAC DKO_luc_cyto (right panel) using 25 μM luciferin. (E) Quantification of changes in ATP levels upon 5 mM glucose addition in BSF 427_Luc_cyto and AAC DKO_luc_cyto. Box and whiskers plots, n = 7–10, *** *P* < 0.001. (F) Representative data of basal (first peak) and glucose-induced (second peak) bioluminescence levels detected by a plate reader in the mitochondrial matrix of BSF 427_luc_mito (left pane) and AAC DKO_luc_mito (right panel) using 25 μM luciferin. (G) Quantification of changes in ATP levels upon 5 mM glucose addition in BSF 427_Luc_mito and AAC DKO_luc_mito. Box and whiskers plots, n = 8–11, *** *P* < 0.001.

To examine whether there is an alternative way for the cytosolic ATP to cross the mitochondrial inner membrane in the absence of AAC, we assayed the capacity of the BSF 427 and AAC DKO mitochondrion to generate a ΔΨ_m_ through the proton-pumping activity of F_o_F_1_-ATP synthase in the presence of external ATP. We permeabilized the *T*. *brucei* plasma membrane with 4 μM digitonin and measured changes in Safranin O fluorescence upon the addition of 1 mM ATP. As Safranine O is imported into the mitochondrion via a ΔΨ_m_ dependent manner, the fluorescent dye undergoes a spectral change that is measured by a fluorimeter. The detected changes in the fluorescence values are used to estimate the ΔΨ_m_ [43]. The control BSF 427 cells were able to create and retain a ΔΨ_m_, as evidenced by a decrease in safranine O fluorescence. Importantly, this quenching of safranine O is completely reversed by the addition of carboxyatractyloside (CATR), the inhibitor of AAC. Subsequent addition of oligomycin before the uncoupler SF 6847 had no further effect on depolarization (Fig 3A, black line). No changes in fluorescence were detected when the addition of CATR preceded that of ATP, confirming that the decrease in safranine O fluorescence is dependent on AAC activity (Fig 3A, red line). Meanwhile, the AAC DKO cell line was unable to generate a ΔΨ_m_ in the presence of external ATP, indicating that no ATP was able to enter the mitochondrial matrix. Importantly, a v5-tagged addback of AAC, expressed from a tubulin gene locus upon the addition of tetracycline, fully rescued the ability to polarize the inner membrane (Fig 3B). Therefore, *in vitro* assays demonstrate that extramitochondrial sources of ATP cannot be imported into the organelle in the absence of AAC.

To confirm the importance of AAC in live cells, we generated reporter BSF 427 and AAC DKO cell lines constitutively expressing a firefly luciferase fused with a C-terminal v5 tag. This heterologous luciferase expression was targeted to either the cytosol (BSF 427_luc_cyto and AAC DKO_luc_cyto) or the mitochondrion (BSF 427_luc_mito and AAC DKO_luc_mito). To promote efficient mitochondrial localization of the luciferase, its gene was fused with the known mitochondrial localization signal of the iron-sulphur cluster assembly protein, ISCU [44]. The expression of the tagged luciferases and their appropriate localization in the cytosol or mitochondrion were verified by western blotting (Fig 3C). Next, we monitored the amounts of ATP in the cytosol of the BSF 427_luc_cyto and AAC DKO_luc_cyto cell lines. The expressed luciferase catalyzes the oxidation of membrane-permeable D-luciferin. This produces bioluminescence proportional to the amount of ATP present in the live cells. Supplementing the buffer of the intact cells with glucose produced an increase in the cytosolic ATP levels in both cell lines, demonstrating the immediate contribution to the cellular ATP pool by glycolysis (Fig 3D). Interestingly, the AAC DKO_luc_cyto cell line had higher levels of ATP compared to BSF 427_luc_cyto parasites (Fig 3E). This discrepancy could be the result of more ATP being sequestered within the cytosol in the absence of mitochondrial ATP import by AAC. Indeed, in the case of cell lines expressing mitochondrially localized luciferase, the addition of glucose caused a spike in mitochondrial ATP levels in the BSF 427_luc_mito cell line, but not in AAC DKO_luc_mito parasites (Fig 3F and 3G). This result verifies that no glucose-derived ATP can be imported into the mitochondrial matrix without AAC.

### The absence of AAC does not significantly alter the proteomic or metabolomic landscapes in BSF *T*. *brucei*

To explore if AAC DKO parasites underwent specific compensatory adaptations that would be reflected by changes in the parasite proteomic landscape, we performed label-free quantitative proteomic analyses. Quadruplicates of BSF 427 and ACC DKO cell lysates were processed using a four-hour liquid chromatography gradient coupled with high-resolution mass spectrometry. The resulting datasets were analyzed by MaxLFQ. We quantified 3,654 protein groups with a minimum of 2 peptides (1 unique) that were present in at least two out of four replicates. Overall, the expression of only 76 proteins was significantly downregulated in AAC DKO, most of which were hypothetical or ribosomal proteins. A total of 44 proteins were significantly upregulated (< 1.5 times, p < 0.05, S1 Fig, S1 Table). From these candidates, only the following proteins were relevant to the scope of this study: succinate dehydrogenase subunit 1 (SDH1, Tb927.8.6580), isocitrate dehydrogenase (IDH, Tb927.8.3690), and amino acid transporter (Tb927.8.8230). Furthermore, some subunits of PDH, KDH, branched-chain ketoamino acid dehydrogenase and mitochondrial pyruvate carrier 2 (MPC2) were also upregulated, although with a *p*-value lower than 0.05 (S1 Fig, S1 Table). Additional mitochondrial enzymes involved in the oxidative metabolism of glucose-derived pyruvate, threonine, and glutamine/glutamate were found unchanged or only slightly affected by the absence of AAC (e.g. malic enzyme (ME)) (S1 Fig). Therefore, we could not identify any obvious known bioenergetic pathways that would indicate that cells lacking AAC need to adapt and activate some compensatory pathways.

In agreement with the proteomics dataset, our metabolomic analysis of the AAC DKO mutant revealed no significant changes in the selected 123 metabolites involved in carbohydrate and amino acid catabolism or in energy metabolism (S2 Table). The only exceptions were some intermediate metabolites of amino acid metabolism that were upregulated, while ribose was strongly downregulated (S1 Fig). In addition, we also detected a slight accumulation of ATP (1.4 times, *p-*value = 0.02) and GTP (1.7 times, *p-*value = 0.008) at the cellular level, suggesting that BSF AAC operates minimally in the reverse mode and in its absence, mitochondrial amino acid metabolism rises marginally. Despite these few observed changes, our results indicate that the AAC DKO mutant does not undergo major restructuring of its global proteomic and metabolomic landscapes in response to the absence of AAC.

### AAC DKO is more sensitive to methyltriphenylphosphonium (TPMP), an inhibitor of α-ketoglutarate dehydrogenase

**Fig 4:**
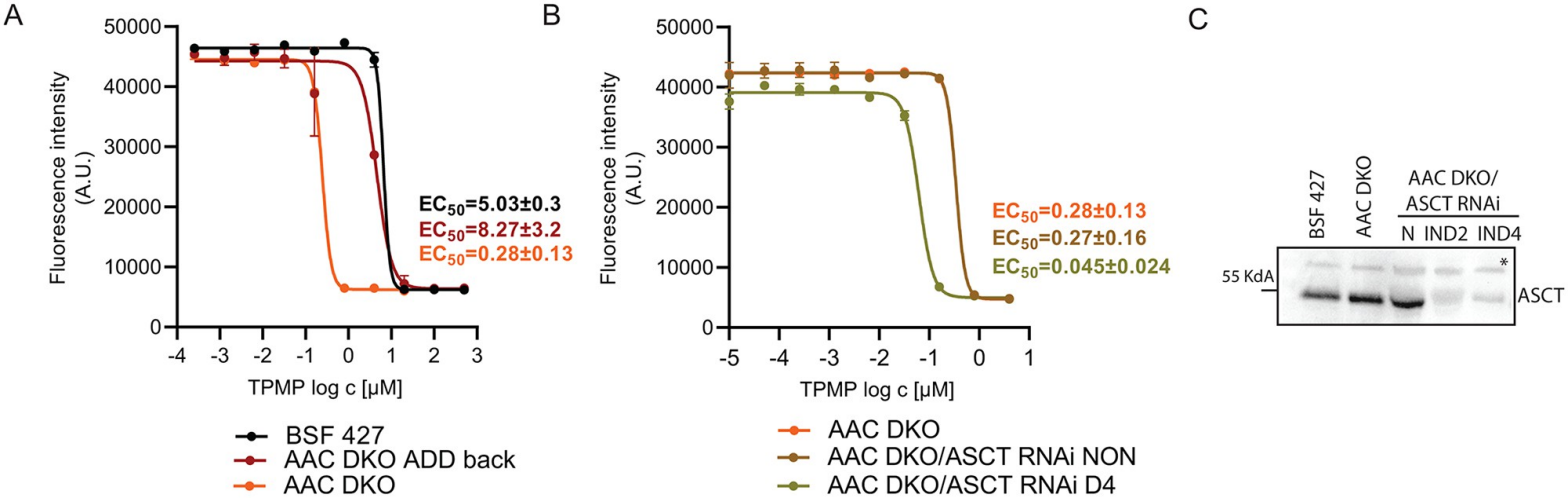
AAC DKO cells are more sensitive to the treatment by TPMP, an inhibitor of α-ketoglutarate dehydrogenase. (A) Sensitivity of BSF 427, AAC DKO, AAC DKO_addback to TPMP estimated by Alamar blue cell viability assay. (B) AAC DKO/ASCT RNAi noninduced (NON) and cells induced for 4 days (D4) to TPMP estimated by resazurine cell-viability assay. The dose-response curves were calculated using GraphPad Prism 8.0 software. The calculated EC_50_ values are shown in graphs and are expressed in μM. (C) Western blot analysis of BSF 427, AAC DKO and AACDKO/ASCT RNAi cells uninduced and induced for 2 and 4 days using anti-ASCT antibody. *-non-specific band serving as a loading control.

The ability of the AAC DKO mutant to maintain the ΔΨ_m_ despite its inability to import cytosolic ATP to the mitochondrial matrix suggests an intramitochondrial source of ATP. This ATP can be generated by substrate-level phosphorylation, with the ATP-producing enzyme SCS being the best candidate (Fig 1). To determine if the ASCT/SCS substrate-level phosphorylation pathway is more important for the AAC DKO mutant, we probed the sensitivity of the AAC DKO and BSF 427 cells to known inhibitors of PDH and TDH. We found no differences in the sensitivity of BSF 427 or AAC DKO cells to the PDH inhibitor, sodium arsenite (BSF 427 EC_50_ = 0.22 μM vs AAC DKO EC_50_ = 0.19 μM). Moreover, the AAC DKO parasites were not more sensitive to any of the TDH inhibitors we applied: quinazolinecarboxamide compound QC1 (BSF 427 EC_50_ = 11.3 μM vs AAC DKO EC_50_ = 9.7 μM) or tetraethyl thiuram disulphide (TETD) (BSF 427 EC_50_ = 9.9 μM vs AAC DKO EC_50_ = 9.4 μM) [45]. Since the PDH and TDH activities are complementary and can compensate for each other [33], it is not surprising that we did not detect any change in the sensitivity of these inhibitors. In contrast, the AAC DKO mutants were 18-fold more sensitive to methyltriphenylphosphonium chloride (TPMP) treatment (Fig 4A), a compound that inhibits KDH [46] (Fig 1). Importantly, AAC DKO parasites expressing an ectopic v5-tagged AAC had TPMP EC_50_ values return to the sensitivity observed in the BSF 427 cell line (Fig 4A). This confirms that the increased significance of KDH is due to the loss of AAC. Because KDH generates succinyl-CoA, the substrate for ATP-producing SCS, the increased importance of KDH activity in the absence of AAC would suggest that the AAC in BSF cells operates to some extent in the reverse mode. Without the normal contribution of AAC to the mitochondrial ATP pool, the parasite is more dependent on mitochondrial ATP substrate-level phosphorylation. The importance of KDH-linked mitochondrial substrate-level phosphorylation is further highlighted by an additional six-fold enhanced sensitivity to TPMP when ASCT expression was suppressed in the background of the AAC DKO parasites (Fig 4B and 4C). Our results suggest that cells lacking AAC are more dependent on the two mitochondrial substrate-level phosphorylation pathways linked by the activity of SCS (Fig 1).

### SCS is expressed and active in the BSF cells

**Fig 5:**
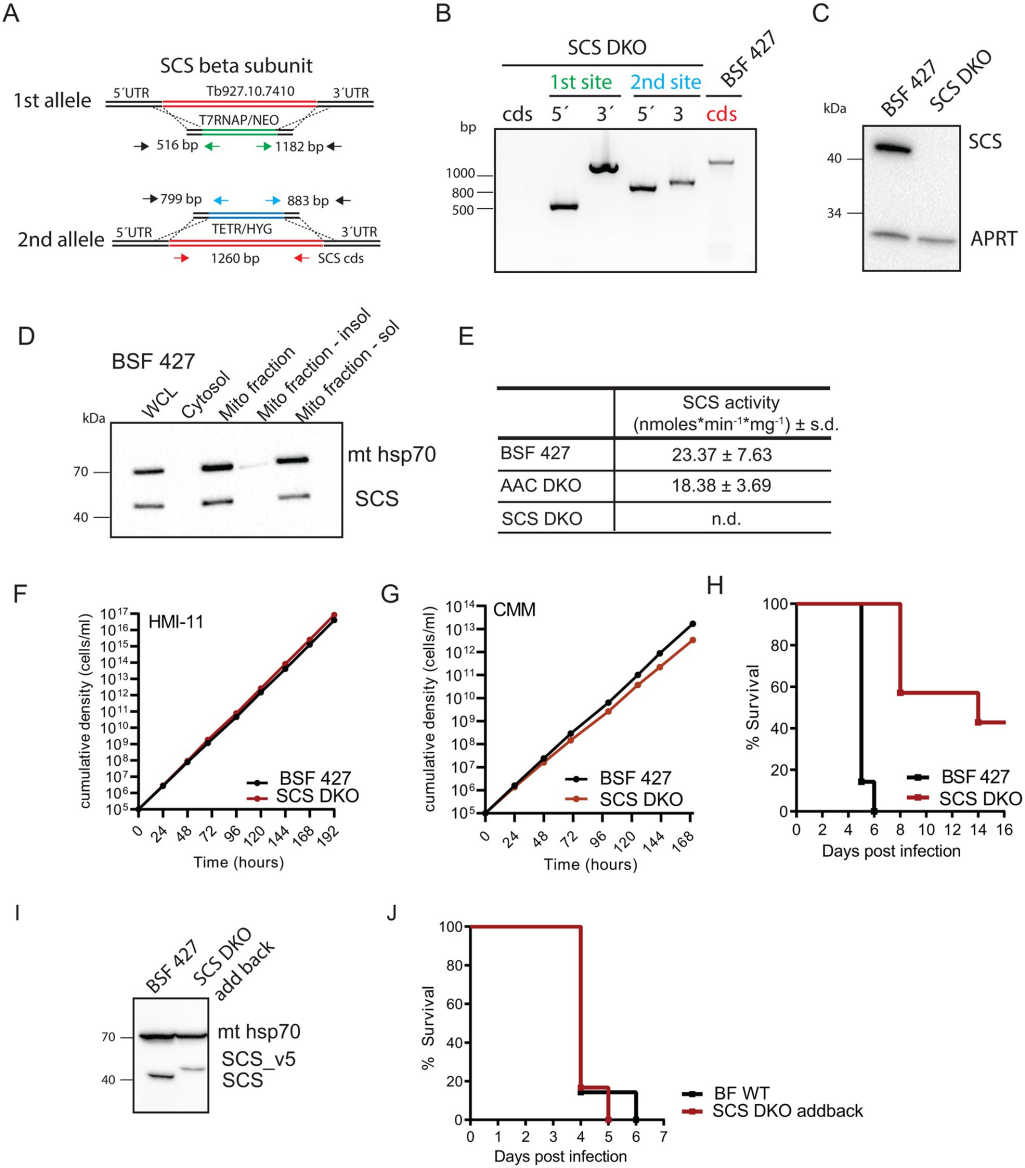
SCS DKO cells are viable *in vitro* but exert lower virulence in animal model. (A) The strategy to generate SCS DKO involved replacement of both alleles with resistance genes conferring neomycin and hygromycin resistance. (B) PCR verification for the elimination of both SCS alleles in SCS DKO cell line. (C) Immunoblot analysis of SCS DKO cells using specific anti-SCS antibody. Immunodetection of cytosolic APRT served as a loading control. (D) Subcellular localization of SCS using BSF 427 cells. WCL, whole cell lysate; Cyt, cytosol; Mito, mitochondrial; insol, insoluble; sol, soluble. (E) Enzymatic activity of SCS measured in mitochondrial lysates extracted from BSF 427, AAC DKO and SCS DKO cells. (F) Growth of AAC DKO cells compared to wild-type BSF 427 in HMI-11 and CMM medium measured for at least 7 days. (G) The survival rate of 7 female BALB/c mice which were intraperitoneally infected with SCS DKO and wild-type BSF 427 parasites. The infected mice were monitored for 14 days. (H) The survival rate of 7 female BALB/c mice which were intraperitoneally infected with SCS DKO Addback and wild-type BSF 427 parasites. The SCS DKO Addback infected mice were supplied with water containing doxycycline to induced expression of the addback SCS copy. The mice were monitored for 6 days. (I) Immunoblot analysis of BSF 427 and SCS cDKO cell line inducibly expressing v5-tagged SCS using specific anti-SCS antibody. Immunodetection of mitochondrial hsp70 served as a loading control. (J) The survival rate of 7 female BALB/c mice which were intraperitoneally infected with BSF 427 and SCS DKO_addback parasites.

SCS is a heterodimer consisting of an α-subunit that binds CoA and a β-subunit that is involved in the generation of ATP. To assess the importance of SCS to the BSF parasites, we generated a double knockout of the ATP-forming β-subunit of SCS (Tb927.10.7410) (Fig 5A). Replacement of both SCS β-subunit alleles with resistance markers was verified by PCR (Fig 5B). The absence of the gene product was further confirmed by Western blot using a specific antibody raised against the recombinant SCS β-subunit (Fig 5C). The SCS enzyme was localized to the mitochondrial matrix as expected (Fig 5D). We also developed an *in vitro* colorimetric assay that measures the amount of CoA produced when the SCS from soluble mitochondrial fractions are incubated with the substrates succinyl-CoA and ADP. The SCS activity measured in both the BSF 427 and AAC DKO cell lines was comparable. Importantly, no SCS activity was detected in the SCS DKO cells, confirming the specificity of this assay and the absence of an alternative gene encoding the β subunit of SCS (Fig 5E).

### SCS DKO parasites display a reduced virulence in a mouse model

The SCS DKO mutants were viable when cultured in both HMI-11 and CMM media (Fig 5F and 5G). To investigate whether SCS is essential for the establishment of infection in animals, we inoculated groups of seven mice with BSF 427 and SCS DKO cells. Mice infected with the control parasites all had to be euthanized for ethical reasons 5–6 days after intraperitoneal injection because a parasitemia of 10^8^ cells/ml was reached. In the case of the SCS null mutants, four of the infected mice were not sick after two weeks and three survived the infection (Fig 5H). The SCS DKO addback cell line (western blot confirmed, Fig 5I) expressing SCS from the tubulin locus was again fully virulent and behaved the same as the BSF 427 parasites. This confirms that the virulence defect was specifically due to the loss of SCS (Fig 5J).

### Metabolomic analysis of SCS mutants reveals changes in the levels of relevant metabolites

To identify possible metabolic changes in SCS DKO trypanosomes at the protein level, we performed quantitative label-free proteomic analyses of SCS DKO whole cell lysates and compared them with BSF 427 samples. Among the 3,654 proteins identified by at least two peptides, only 17 and 21 proteins were up- or down-regulated by more than 1.5-fold, respectively, in the mutant cell line (p < 0.05). This corresponds to approximately just 0.5% of the proteome. Due to the small size of significantly altered hits, the GO ontology enrichment analyses did not reveal any enrichment of GO term categories (S2 Fig, S1 Table). Notably, one of the downregulated proteins was also SCS subunit α, presumably due to the lack of its heterodimer partner, subunit β. In conclusion, similar to AAC DKO, there is no major remodeling of metabolic pathways in the SCS DKO mutant that would be reflected by a change in the steady-state abundance of their enzymatic components.

We also performed a targeted metabolomic analysis of the SCS DKO mutant and BSF 427 parasites. Of the 127 metabolites analyzed, we found an enrichment of acetyl-CoA (2.4-fold) and α-ketoglutarate (1.5-fold). This finding demonstrates the veracity of the metabolomics approach, as these metabolites lie just upstream of the two different substrate-level phosphorylation pathways that depend on SCS. Furthermore, the TCA cycle metabolites (succinate, fumarate and malate) that are synthesized immediately downstream of SCS were all strongly downregulated. Interestingly, we also observed increases of oxaloacetate (5.4-fold) and glutamate (1.6-fold), a possible source of α-ketoglutarate, along with decreased levels of aspartate (0.6-fold). This finding suggests a downregulation of the mitochondrial aspartate aminotransferase, an important enzyme in amino acid metabolism that is normally expressed in BSF cells. The aspartate aminotransferase is a key enzyme in the malate-aspartate shuttle, which is a biochemical system designed to balance the levels of mitochondrial and cytosolic NADH. Alterations were also observed in metabolites belonging to the pentose phosphate pathway and amino acid metabolism (S2 Fig, S3 Table). Importantly, our analysis doesn’t distinguish between glycosomal, cytosolic, and mitochondrial dicarboxylic acid pools. It’s unlikely that the SCS absence affects the glycosomal succinate reduction pathway, which functions mainly as a redox balancer for glycolysis. Nevertheless, more metabolomic studies with labeled-carbon sources (e.g., glucose, glutamine) are needed to pinpoint the metabolites’ exact origin and location.

### The excretion of acetate is abolished in the SCS DKO mutant

**Fig 6:**
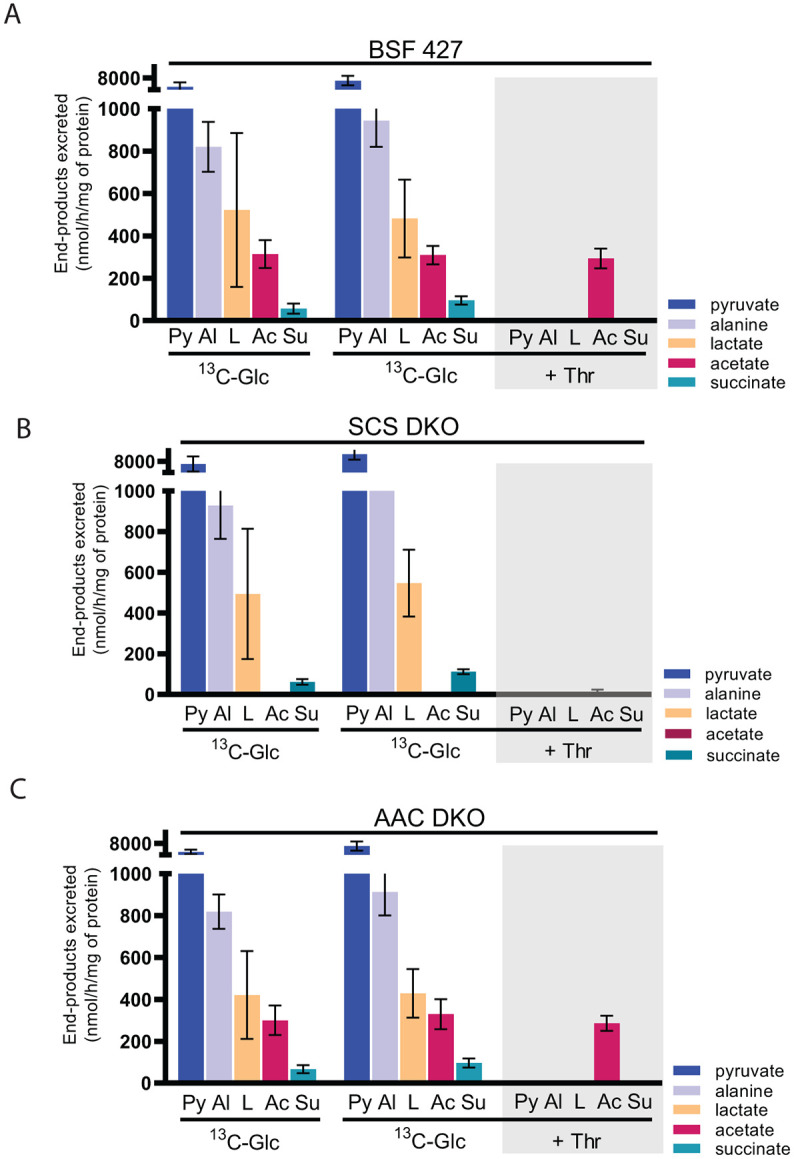
SCS DKO parasites do not excretes acetate. Proton (^1^H) NMR analyses of end-products excreted from the metabolism of ^13^C-enriched glucose. BSF 427 (A), SCS DKO (B) and AAC DKO (C) trypanosomes were incubated for 2.5 hours in PBS containing 4 mM [U-^13^C]-glucose in combination with threonine (+Thr) or α-ketoglutarate (+α-KG) before analysis of the spent medium by ^1^H-NMR spectrometry. The amounts of each end-product excreted are documented in S3 Table. Abbreviations: Ac, acetate; Al, alanine; L, lactate; Py, pyruvate; S, succinate.

Next, we wanted to closely examine if either of the SCS substrate-level phosphorylation pathways contribute to the levels of known BSF excreted metabolic end-products. Therefore, we incubated BSF 427, SCS DKO and AAC DKO cells in [U-^13^C]-enriched glucose-containing PBS and used ^1^H NMR spectrometry to quantify the amount of ^13^C-enriched end-products produced via glucose metabolism that were excreted into the medium. BSF 427 cells excreted predominantly high amounts of pyruvate (79.8%) and to a lesser amount alanine (10.6%), lactate (4.1%), acetate (3.9%) and succinate (1.6%) (Fig 6A, left panel). Analysis of the SCS DKO mutant revealed that there were no significant changes in the excretion of pyruvate (82.8%), alanine (10.7%), succinate (1.2%), and lactate (5.0%). However, the excreted acetate derived from glucose was completely abolished (Fig 6B, left panel). Since acetate can also be produced from threonine, we utilized an alternative ^1^H NMR spectrometry method in which each cell line was incubated with equal amounts (4 mM) of both uniformly [^13^C]-enriched glucose and unenriched threonine to distinguish the metabolic origin of the excreted acetate (Fig 6A and 6B, middle and right panel). Interestingly, acetate excretion from both [U-^13^C]-enriched glucose and unenriched threonine was almost abolished in the SCS DKO cell line, with only residual amounts of threonine-derived acetate detected. This confirms that the ASCT/SCS cycle coupled to ATP generation is the primary source of acetate that is excreted. Because SCS DKO cells did not exhibit a growth phenotype in either HMI-11 or CMM medium, we suggest that the activity of ACH in SCS DKO cells maintains the intracellular acetate levels necessary for *de novo* biosynthesis of fatty acids in the absence of SCS (Fig 1) [4]. Unlike the SCS DKO mutant that resulted in significant metabolic changes, we did not observe any changes in glucose- and threonine-derived metabolic end products in the AAC DKO cell line (Fig 6C, S4 Table). This is consistent with the metabolomic data (S1 Fig, S2 Table), suggesting that the absence of AAC is well tolerated by the BSF cells as mitochondrial substrate-level phosphorylation pathways are able to fully compensate for its loss.

### SCS DKO parasites have reduced mitochondrial ATP levels, but maintain normal levels of ΔΨ_m_

**Fig 7:**
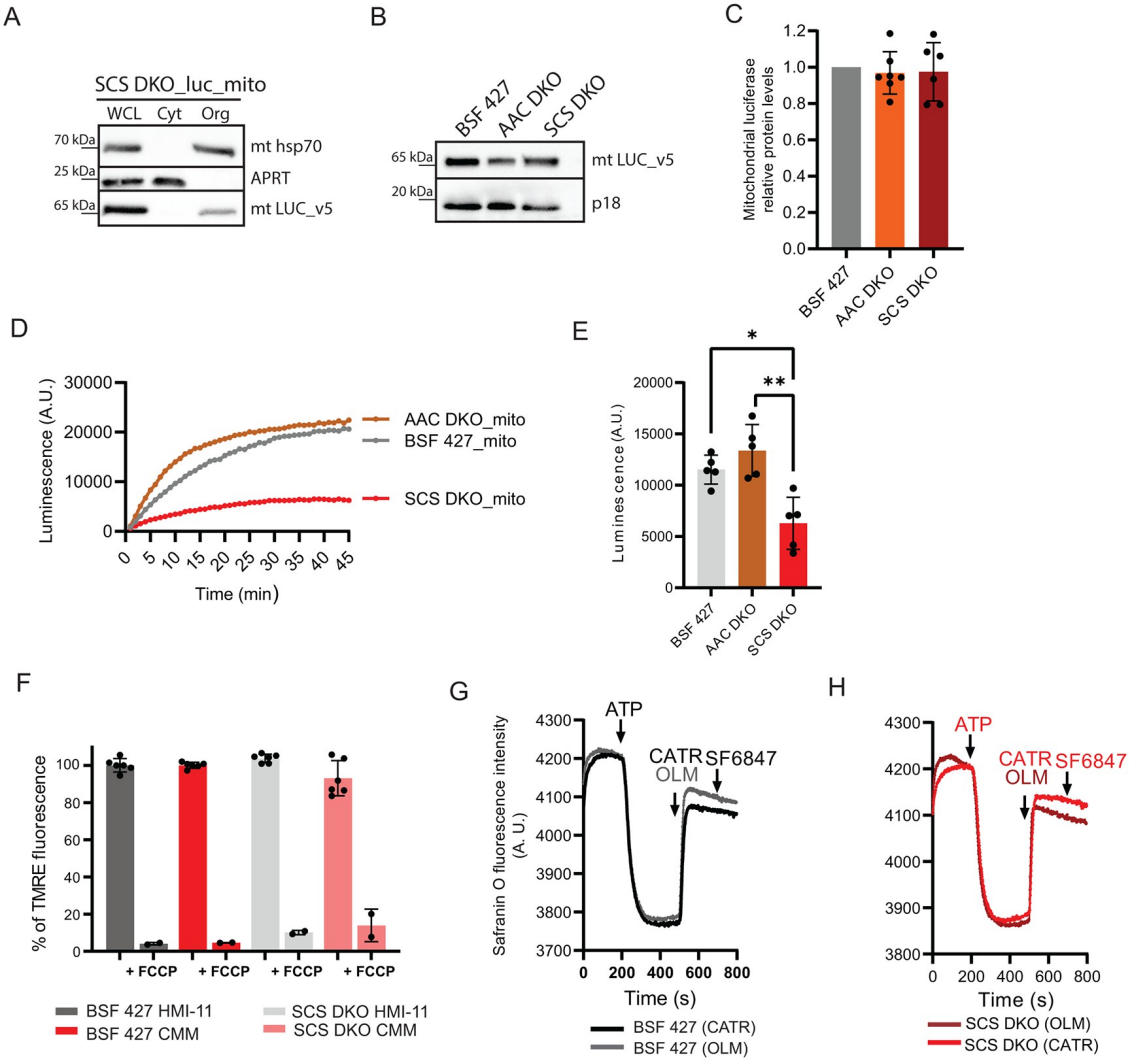
SCS DKO parasites have decreased mitochondrial ATP content, but are capable of ATP import and ATP hydrolysis. (A) Subcellular localization of V5-tagged luciferase with mitochondrial localization signal (luc_mito) endogenously expressed in SCS DKO cells was determined in whole cell lysates and in the corresponding cytosolic and organellar fractions separated by digitonin extraction. Purified fractions were analyzed by Western blotting with the following antibodies: anti-v5, anti-mt Hsp70 (mitochondrial marker), and anti-adenosine phosphoribosyltransferase (APRT) (cytosolic marker). The relevant sizes of the protein marker are indicated on the left. (B) Immunoblot of V5-tagged luciferase expressed in BSF 427_luc_mito, AAC DKO_luc_mito and SCS DKO_luc_mito cells using antibodies against V5 tag. Antibody against subunit p18 of F_o_F_1_ ATP synthase was used as a loading control. (C) The quantification analyses of luciferase expression in all three cell lines by densitometry. The bars represent relative protein amounts of luciferase expression in AAC DKO and SCS DKO cells compared to luciferase expression in BSF 427. (means ± s.d., n = 6–7). (D) Representative data of ATP measurements performed in living BSF 427_luc_mito, AAC DKO_luc_mito and SCS DKO_luc_mito cells using 25 μM luciferin. (E) Quantification of the luminescence measurement detected in BSF 427_luc_mito, AAC DKO_luc_mito, SCS DKO_luc_mito. Data shown in the bars are derived from experiments of which representative graphs are shown in panel D (means ± s.d., n = 5, Student´s unpaired *t*-test, *P < 0.05, ** *P* < 0.005). (F) Flow cytometry analysis of TMRE-stained SCS DKO and BSF 427 cells grown in HMI-11 or CMM medium to measure ΔΨm. The addition of FCCP served as a control for ΔΨ_m_ depolarization (+FCCP). (means ± s.d., n = 6). (G, H) Mitochondrial membrane polarization detected using Safranine O dye in digitonin-permeabilized BSF 427 cells (black/grey lines) and SCS DKO (light and dark red) in the presence of ATP. ATP, CATR, OLM and SF 6847 were added where indicated.

To further explore the contributions of mitochondrial substrate-level phosphorylation in BSF parasites, we measured the mitochondrial ATP levels in SCS DKO parasites and compared them with BSF 427 and AAC DKO cells. For this purpose, we generated SCS DKO cells constitutively expressing a mitochondrial v5-tagged luciferase. The expression and localization of the mitochondrial luciferase was verified in the same manner as the mitochondrial luciferase-expressing BSF 427 and AAC DKO cells (Figs 2C and 7A). In all three cell lines, the mitochondrial luciferase expression levels were comparable, without any statistically significant differences. This consistency in enzyme expression allowed us to compare mitochondrial ATP levels between the different cell lines (Fig 7B and 7C). Unlike the previous experiments in Fig 2D–2G, where we measured the dynamics of glucose-induced ATP production, we now measured the intramitochondrial steady-state ATP levels of the cell lines. The reaction was initiated by the addition of D-luciferin. As the luciferin enters the cell, the amount of emitted light will rapidly increase until it reaches a plateau after a certain amount of time depending on the cell line (Fig 7D). The luminescence emissions at the plateau for each cell line from numerous independent experiments were plotted as a column graph (Fig 7E). The mitochondrial ATP levels in BSF 427 and AAC DKO reached similar levels. Knowing that the mitochondrion of AAC DKO is not capable of importing ATP, this ATP pool must be produced intramitochondrially. Importantly, statistically less ATP was detected in the mitochondrial matrix of the SCS DKO mutant cells, when compared to AAC DKO and BSF 427 cells (Fig 7E). Without the possibility to produce ATP by substrate-level phosphorylation, it is likely that this ATP pool is generated by the reverse activity of AAC.

The reverse AAC activity is apparently capable of providing a sufficient amount of ATP to maintain the ΔΨ_m_, as no significant difference was detected in the fluorescence intensity of TMRE-stained BSF 427 and SCS DKO cell populations grown in either HMI-11 or CMM medium (Fig 7F). Furthermore, the ATP-induced polarization of the mitochondrial inner membrane in SCS DKO digitonin-permeabilized cells followed the same pattern as in BSF 427 cells, suggesting that AAC is able to import ATP into the mitochondrion and this ATP is used to energize the membrane using F_o_F_1_-ATP synthase (Fig 7G and 7H).

### SCS DKO cells are dependent on ATP import from the cytosol

**Fig 8:**
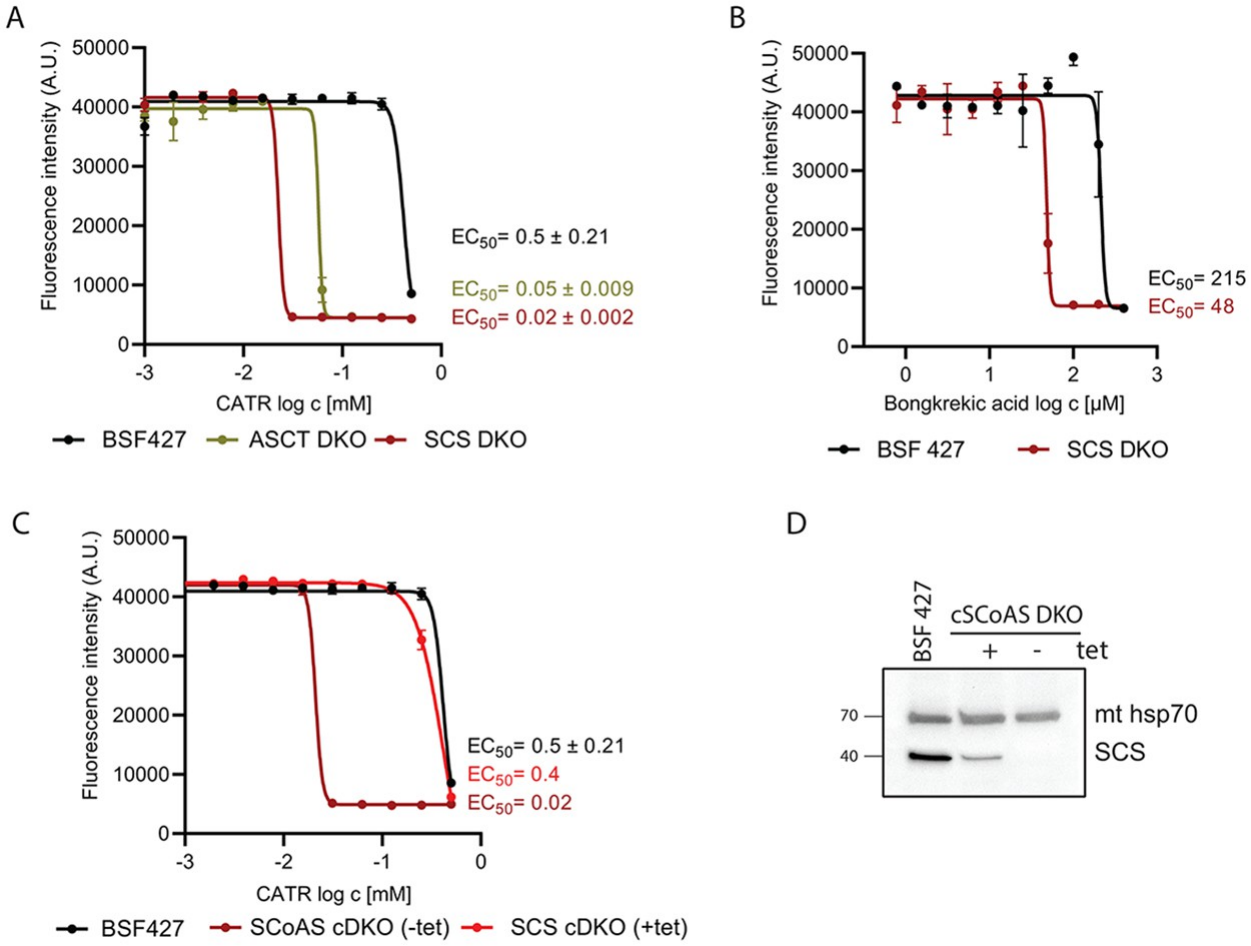
SCS DKO cells are more sensitive to CATR, an inhibitor of AAC. (A) Sensitivity of BSF 427, SCS DKO, ASCT DKO to carboxyatractyloside (CATR) estimated by Alamar Blue cell-viability assay. The dose-response curves were calculated using GraphPad Prism 8.0 software. The calculated EC_50_ values are shown in graphs and are expressed in mM. (B) Sensitivity of BSF 427 and SCS DKO, ASCT DKO to bongkrekic acid estimated as in (A). (C) Sensitivity of BSF 427, SCS cDKO noninduced (-tet) and 4-days induced (+tet) cells to carboxyatractyloside (CATR) estimated as in (A). (D) Immunoblot of SCS cDKO noninduced (-tet) and 2-days induced (+tet) cells using SCS antibody. Immunodetection of mitochondrial hsp70 served as a loading control.

If mitochondrial substrate-level phosphorylation pathways contribute to the BSF mitochondrial ATP pool under normal physiological conditions, then the SCS DKO cell line must implement a compensatory mechanism to maintain the ΔΨ_m_. Therefore, we examined if SCS DKO parasites become more dependent on the reverse AAC activity to import the necessary ATP into the mitochondrial matrix. Indeed, an SCS DKO cell viability assay demonstrated that these mutants are more sensitive to CATR and bongkrekic acid, both of which are specific inhibitors of AAC. Compared to BSF 427, the EC_50_ values of SCS DKO parasites were ~25-fold lower in the case of CATR and ~5-fold lower for bongkrekic acid (Fig 8A and 8B). Consistent with this observation, the ASCT DKO cell line, which is defective in only one of the two mitochondrial substrate-level phosphorylation pathways, demonstrated only a 10-fold higher sensitivity to CATR compared to BSF 427 (Fig 8A). Tetracycline-induced expression of SCS in the background of the SCS null mutant restored the original EC_50_ values for CATR, confirming that the observed phenotype in CATR-sensitivity was due to the absence of SCS (Fig 8C and 8D).

In summary, it appears that *T*. *brucei* BSF parasites have two alternative options for mitochondrial ATP provision, intramitochondrial ATP production by substrate-level phosphorylation and ATP import from the cytosol via AAC. To test the essentiality of this intriguing functional interplay, we attempted to silence AAC expression by RNAi in the SCS DKO background, while also conversely pursuing to silence SCS expression in the AAC DKO background. Unfortunately, our numerous attempts failed to generate selected transfectants that retained a robust tetracycline-induced silencing of the targeted gene. While an extremely unsatisfactory result, we interpret this phenomenon to indicate that any expression of dsRNA in the absence of tetracycline during the selection process causes a lethal phenotype. Therefore, it is likely that these two pathways act complementarily to each other and the absence of both is not consistent with the survival of *T*. *brucei* parasites under the conditions used.

### Mitochondrial production of ATP by substrate-level phosphorylation is essential under glycerol-rich growth conditions

**Fig 9:**
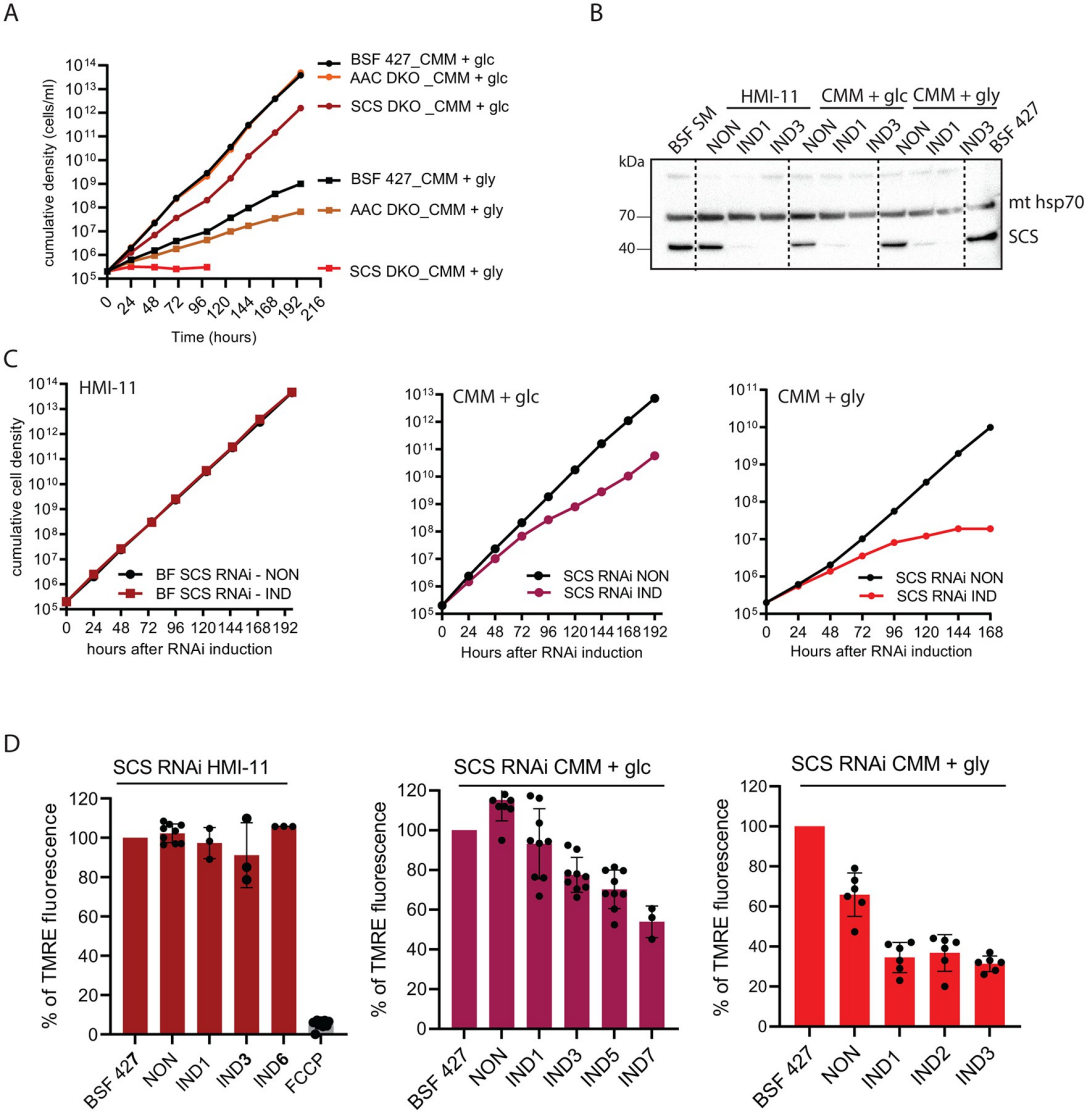
SCS RNAi silencing results in growth phenotype and decreased ΔΨ_m_ in CMM_glc and CMM_gly medium. (A) Growth of BSF 427, AAC DKO and SCS DKO cells in CMM_glc and CMM_gly medium. (B) Western blot analysis of whole cell lysates of SCS RNAi non induced and induced (+tet) cells grown in HMI-11, CMM_glc and CMM_gly using antibodies against the SCS protein. The immunoblot probed with anti-mitochondrial hsp70 antibody served as loading controls. Glc, glucose; gly, glycerol. (C) Growth of SCS RNAi noni nduced (non) and tetracycline induced (IND) cells measured for 8 days in HMI- 11 (left), CMM_glc (middle) and CMM_gly (right). Glc, glucose; gly, glycerol. (D) Flow cytometry analysis of TMRE-stained SCS RNAi noninduced and induced cells grown in HMI- 11 (right), CMM_glc (middle) and CMM_gly (left). (means ± s.d., n = 3–9).

In addition to glucose, the dividing BSF can also use glycerol as an energy and carbon source [47,48]. Nevertheless, the growth rate of BSF 427 is significantly reduced when grown in glycerol-rich (10 mM) medium (CMM_gly) (Fig 9A). This is due to the limited capacity of BSF *T*. *brucei* to metabolize glycerol compared to glucose, which results in a slightly lower yield of cytosolic ATP compared to cells grown in CMM medium containing 10 mM glucose (CMM_glc). Interestingly, BSF grown in CMM_gly excrete more acetate and succinate than those grown in CMM_glc, suggesting a higher activity of these mitochondrial metabolic pathways [47]. Therefore, we further investigated the importance of SCS for cells grown in CMM_gly. The BSF 427 and AAC DKO cells were able to adapt to glycerol conditions well, albeit they grow with a slower doubling rate. In contrast, the SCS DKO parasites were never able to establish an adapted culture that actively divided (Fig 9A). Therefore, we wanted to determine if lower cytosolic ATP yields further reduced the rate of AAC to import ATP into the mitochondrion. This would require BSF *T*. *brucei* grown in glycerol media to rely more on mitochondrial substrate-level phosphorylation to provide its own pool of ATP for the F_1_F_o_-ATP synthase to hydrolyze and maintain the ΔΨ_m_. To investigate the primary effect of SCS depletion, we generated RNAi cells to silence SCS expression of cultures grown in either HMI-11, CMM_glc or CMM_gly. The efficiency of the RNAi-mediated downregulation of SCS was verified under all three growth conditions by Western blot using specific antibodies (Fig 9B). The propagation of the SCS RNAi cell line grown in HMI-11 medium was not affected by the addition of tetracycline (Fig 9C). Furthermore, we did not detect any decrease in the ΔΨ_m_ by flow cytometry in TMRE-stained noninduced and tetracycline-induced cells (Fig 9D). However, silencing of SCS in CMM_glc medium resulted in an even longer doubling time of the RNAi-induced cell population. Compared with BSF 427 cells, the ΔΨ_m_ was decreased by approximately 30% in cells induced for 5 days, which most likely contributed to the mild growth phenotype of these cells. Most importantly, SCS RNAi cells grown in CMM_gly exhibited a severe growth phenotype associated with a sharp decrease in ΔΨ_m_ at days 1, 2, and 3 after induction (Fig 9C and 9D). In this case, ΔΨ_m_ values fell below the minimum threshold required for *T*. *brucei* viability *in vitro* [22,28].

Our data clearly indicate that the functional interplay between AAC and an ATP-producing SCS depends on the environment. When the parasites encounter environments with lower glucose concentrations or other carbon sources (e.g. glycerol) that yield lower cytosolic ATP levels, the BSF *T*. *brucei* relies on mitochondrial substrate-level phosphorylation pathways as it cannot augment its mitochondrial ATP pools by using the reverse AAC activity to withdraw ATP from the cytosol. This is probably also true when the parasites multiply in the bloodstream of their mammalian host, where they are exposed to various challenges and are therefore likely to consume greater quantities of cytosolic ATP. In agreement with this, the SCS DKO mutants are less virulent in the mouse model. The ability of AAC to reverse its activity depends on the levels of ΔΨ_m_, cytosolic ATP levels and the ATP/ADP ratio in the mitochondrial matrix. Therefore, the parasite bioenergetics regulates the major contributing pathways of ATP provision that are fully compensatory when the parasite is in glucose-rich HMI-11 culture conditions. However, when in *in vivo* or CMM_gly culture conditions, our data show that the mitochondrial substrate-level phosphorylation pathways become more important for parasite survival. From all the data together, we can conclude that the BSF mitochondrion is capable of producing ATP.

## Discussion

Historically, it was purported that maintenance of the *T*. *brucei* long-slender BSF ΔΨ_m_ occurred via the proton-pumping F_o_F_1_-ATP synthase hydrolyzing mitochondrial ATP imported from the cytosol by the reverse activity of AAC. This notion that the organelle only consumes ATP has persisted through the *T*. *brucei* literature for decades. However, the widespread assumption that the long slender BSF possesses a simplified mitochondrion has recently been challenged by proteomic and metabolomic data suggesting that certain metabolic pathways can be activated under permissible environmental conditions [11]. High flexibility and adaptability of the parasite organelle can be beneficial for the parasite when adapting to new host environments (e.g. when populating adipose tissue or skin) [49,50]. Indeed, the discovery of a mitochondrial acetate production pathway that is linked to an ATP-producing activity, has questioned this classical view [33,51,52].

The confounding factor of the *T*. *brucei* BSF ΔΨ_m_ centers on the directionality of AAC. It has been clearly demonstrated that due to the cellular conditions (mitochondrial matrix ATP/ADP ratio and ΔΨ_m_), the BSF F_o_F_1_-ATP synthase has reached its reversal potential (E_rev_ATPase_) and hydrolyzes ATP to generate a ΔΨ_m_ between -150 to -180 mV [15,16]. Intriguingly, mathematical modeling of mammalian mitochondria undergoing mitochondrial membrane depolarization induced by ETC inhibition or hypoxia showed that the F_o_F_1_-ATP synthase first reaches its E_rev_ATPase_ value before the conditions also dictate AAC reversal. Under this circumstance, mitochondrial ATP is generated by mitochondrial substrate-level phosphorylation. If the ΔΨ_m_ shifts to even less negative values or the balance between the ATP/ADP ratio in the mitochondrial matrix and the ATP levels in the cytosol is further disturbed, then AAC will also reverse (E_rev_AAC_) [19]. These mathematical models therefore suggest that while the BSF F_o_F_1_-ATP synthase operates in the reverse mode, this activity may not be dependent on the reverse mode of AAC. Indeed, we demonstrate that AAC DKO *T*. *brucei* BSF cells are viable *in vitro* and fully virulent in a mouse model, suggesting that the import of cytosolic ATP into the mitochondrion is dispensable.

**Fig 10:**
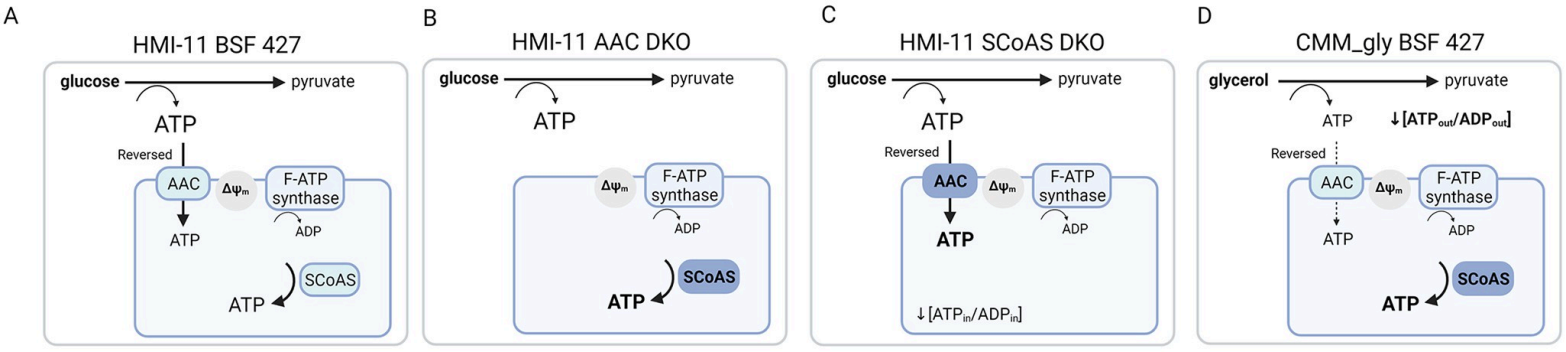
Schematic visualization of AAC and SCS activities interplay in BSF 427 (A), AAC DKO (B) and SCS DKO (C) grown in HMI-11 and BSF 427 cultured in CMM_gly medium (D). AAC, ATP/ADP carrier; SCS, succinyl-CoA synthetase.

Our data also clearly show that AAC is the only carrier that can import ATP into the mitochondrial matrix since the addition of high ATP concentrations (1 mM) did not induce mitochondrial membrane polarization in the AAC DKO parasites with permeabilized plasma membranes. To demonstrate this phenomenon in another way, we treated intact BSF 427 cells resuspended in a defined buffer with external glucose to stimulate cytosolic generations of ATP. Since these cells were engineered with luciferase targeted to the mitochondrion, we detected that this ATP was imported into the mitochondrial matrix. However, there was no detected increase in the mitochondrial ATP levels in the AAC DKO mutants also expressing mitochondrial luciferase. Therefore, in the case of the AAC DKO parasites, the standard culture medium and host environment must provide enough nutrients to support mitochondrial ATP production by mitochondrial substrate-level phosphorylation pathways that are powerful enough to provide sufficient amounts of ATP to maintain the ΔΨ_m_ at levels compatible with full parasite virulence (Fig 10B).

Although the metabolomic changes in the AAC DKO parasites may indicate to some extent the higher activity of mitochondrial metabolic pathways linked to ATP production, the determined levels of metabolic end-products (i.e. pyruvate, acetate, succinate and alanine) showed no significant changes. This lack of a striking metabolic remodeling suggests that in the absence of AAC, the BSF mitochondrion is capable of being fully independent of the cytosolic supply of ATP. Therefore, it seems that AAC does not operate at a high rate in the reverse mode under physiological conditions (no striking phenotype in AAC DKO cells) (Fig 10A and 10B). However, it appears that AAC can increase the rate of mitochondrial ATP import, whenever the mitochondrial substrate-level phosphorylation pathways are not able to provide enough ATP. High levels of the cytosolic ATP allow for an immediate reversal of AAC, in which the cytosolic ATP pool contributes to the ΔΨ_m_. This is exemplified in the SCS DKO cell line, where the mitochondrial ATP/ADP ratio is decreased. Indeed, our luciferase-based assay showed lower mitochondrial steady-state ATP levels in the SCS DKO parasites when compared to BSF 427 and AAC DKO cells. Since we measured the same ΔΨ_m_ values in each of these cell lines (Fig 10C), the AAC activity is fully reversed to import ATP in compensation for the loss of SCS (Fig 10C). The increased dependence on the reverse rate of AAC is evident by the dramatic increase in sensitivity to the AAC inhibitor, CATR.

Based on the metabolic pathways mapped to the parasite mitochondrion [31–33], there are at least two options for generating the succinyl-CoA substrate required for the ATP-producing SCS. First, succinyl-CoA can be produced by ASCT enzyme from the pyruvate- and threonine-derived acetyl-CoA. The importance of the ASCT/SCS cycle for the ATP/ADP ratio in mitochondrial matrix is supported by the observation that ASCT DKO parasites are 10-times more sensitive to CATR compared to BSF 427. Originally, the metabolic pathways leading to the production of acetyl-CoA were studied from the point of acetate production, the essential precursor for *de novo* fatty acid biosynthesis [53]. Indeed, when both pathways leading to acetyl-CoA were genetically impaired, growth of BSF cells in HMI-11 medium is strongly affected because of the inability to produce acetate [33]. Interestingly, excretion of acetate was greatly reduced but not fully abolished in SCS DKO, suggesting that this baseline level of acetate production is due to the ACH activity and sufficient to support fatty acid biosynthesis without affecting parasite growth rate [5,7,54].

The second potential source of succinyl-CoA could be derived from α-ketoglutarate by KDH, an enzyme with an enigmatic function in BSF cells [55]. The α-ketoglutarate can be derived from glutamine, an amino acid that is consumed in significant amounts [31]. Another possible source of α-ketoglutarate are the transamination reactions employing alanine and aspartate aminotransferases. Alanine aminotransferase is probably essential for BSF cells, as BSF parasites excrete significant amounts of alanine from pyruvate. Although it is a cytosolic enzyme [56,57], the Tryptag data places this enzyme also into the mitochondrial matrix [58,59]. In addition, α-ketoglutarate should also be considered a potentially excellent external source of carbon, as recently observed for PCF trypanosomes [5].

In conditions when glycerol is the main carbon source, the BSF parasites can utilize it in a similar manner as glucose by converting it into pyruvate, alanine, acetate and succinate. To produce similar amounts of cytosolic ATP, twice as much glycerol (a three-carbon compound) must be metabolized as glucose (a six-carbon compound). However, the BSF 427 strain consumes only 1.5-times more glycerol than glucose when grown in CMM_gly and CMM_glc, respectively. This explains the significant growth delay observed in parasites cultivated in CMM_gly [47]. Interestingly, the absolute amounts of acetate produced in CMM_gly and CMM_glc is similar (283 *versus* 262 nmol/h/10^8^ cells) [47]. This suggests that maintaining mitochondrial substrate-level phosphorylation is important, especially when cytosolic ATP is reduced as expected in CMM_gly. This hypothesis is in agreement with the observation that the SCS DKO cells were not able to establish a proliferative culture in CMM_gly medium and the SCS RNAi induced cells exhibited a strong growth retardation followed by a significant decrease of ΔΨm. Indeed, the glycerol-induced reduction of the cytosolic ATP levels may create conditions under which the reverse AAC activity is no longer sufficient to compensate for the absence of mitochondrial substrate-level phosphorylation (Fig 10D). Alternatively, we cannot exclude that the reverse AAC activity significantly depletes the cytosolic ATP pool to levels that are detrimental to the parasite.

In summary, we can conclude that BSF *T*. *brucei* exhibit an amazing flexibility in its cellular bioenergetics, which enables the parasite to quickly adapt and survive various challenging environments of its mammalian host by responding to sudden changes in intracellular ATP levels while still maintaining viable levels of the ΔΨ_m_ across the mitochondrial inner membrane.

## Material and methods

### Trypanosoma cultures

*T*. *brucei brucei* bloodstream Lister 427 form (BSF 427) and genetic derivatives thereof were used in this study. The long slender monomorphic BSF were cultured in HMI-11, Creek Minimal Medium (CMM) containing 10 mM glucose (CMM_glc) or 10 mM glycerol (CMM_gly) supplemented with 10% heat-inactivated fetal bovine serum (FBS) at 37°C in the presence of 5% CO_2_. The genetically modified parasites were cultivated in HMI-11 medium in the presence of appropriate antibiotics to maintain their genetic background (G418 in 2.5 μg/ml, hygromycin in 5 μg/ml, puromycin in 0.1 μg/ml, phleomycin in 2.5 μg/ml, and tetracycline in 1 μg/ml). When needed, the cells were transferred to CMM_glc or CMM_gly media and maintained for two weeks before any experiments were performed (except for the experiment shown in Fig 9A). The cells were always kept in a logarithmic growth phase and harvested at a density of 0.7–1.4 x 10^6^ cells/ml.

### Plasmids and generation of genetically modified cell lines

The AAC double knock-out (DKO) and SCS DKO were generated by two rounds of homologous recombination using gene knock-out (KO) cassettes conferring either neomycin (G418) or hygromycin resistance. The gene cassettes were derived from the pLEW13 and pLEW90 vectors, respectively [60]. To direct the allele replacement, the KO casettes were flanked by short sequences of either AAC (Tb92710.14820/-30/-40) or SCS subunit β (Tb927.10.7410) 5´and 3´ untranslated regions (UTR) that were identified with TritrypDB. The UTR fragments were amplified by PCR from BSF 427 genomic (g)DNA with 5´UTR_forward and reverse or 3´UTR_forward and reverse primers (S5 Table). The amplicons were then digested with Not I and MluI restrictions enzymes (5´UTRs) or XbaI and StuI (3´UTRs) before sequentially ligated into the pLEW13 plasmid that contains genes for neomycin-resistance and T7 RNA polymerase gene. The final pLEW13_AAC_5´/3´UTRs and pLEW13_SCS_5´/3´UTRs constructs were linearized with Not I and electroporated with human T cell nucleofector solution (AMAXA) into BSF 427 to generate a single KO cell line. The transfected cells were serially diluted after 16 hours of recovery and selected with 2.5 μg/ml G418. To generate the double knock-out, the hygromycin-resistance cassette containing the tetracycline repressor under T7 RNAP promoter was excised from the pLEW90 vector with XhoI and StuI restriction enzymes and used to replace the neomycin-resistance cassette from the pLEW13_AAC_5´/3´UTRs and pLEW13_SCS_5´/3´UTRs construct pre-digested with XhoI and SwaI endonucleases. The AAC_ and SCS_ single knock-out cells were transfected with the NotI-linearized plasmids followed by selection using hygromycin (5 μg/ml). AAC DKO and SCS DKO were grown in the presence of 2.5 μg/ml G418 and 5 μg/ml hygromycin.

To downregulate expression of SCS, DNA fragment corresponding to 591 bp target sequence was amplified by PCR from BSF 427 gDNA using gene forward and reverse primers (S5 Table) extended with BamHI and HindIII restriction sites. The resulting PCR product was digested with the corresponding enzymes and inserted into digested p2T7-177 plasmid [61]. The single marker BSF 427 cell line, which bears cassettes for T7 RNAP and tetracycline repressor under neomycin-resistance marker allowing for inducible expression of dsRNA using tetracycline, was transfected with the NotI-linearized plasmid. SCS RNAi were kept in G418 and phleomycin again with induction of RNAi by tetracycline.

To generate constructs for the constitutive expression of luciferase targeted to either cytosol or mitochondrial matrix, the luciferase gene was amplified by PCR using gene specific forward and reverse primers. To ensure mitochondrial localization of the luciferase, the mtLuc_FW primer was extended on its 5´termini with TbIscU mitochondrial targeting sequence [44]. The amplified *luc_mito* and *luc_cyto* genes were digested with BamHI and HindIII restriction enzymes and cloned into the modified pHD1344-tub-B5-3v5 vector (provided by J. Carnes and K. Stuart) that was pre-digested with the same enzymes to remove the original gene for TbKREPB5. BSF 427, AAC DKO and SCS DKO cells were transfected with the final NotI-linearized plasmids pHD1344-tub-mtLUC-3v5 and pHD1344-tub-cytLUC-3v5. The integration into the tubulin locus ensures constitutive expression of the gene of interest. Luciferase cell line in the BSF 427 background was grown in puromycin and in the background of AAC DKO and SCS DKO cells were grown in G418, hygromycin, and puromycin.

The AAC DKO/ASCT RNAi cell line was generated by transfecting Not1-linearized pLew-ASCT-SAS construct containing N-terminal fragment of *asct* gene [62] to AAC DKO cell line. AAC DKO/ASCT RNAi were grown in G418, hygromycin, and phleomycin with the induction of RNAi by tetracycline.

AAC DKO and SCS DKO addback cell lines were generated in the background of the respective DKO cells. The coding sequences of AAC and SCS were amplified from BSF 427 gDNA using specific forward and reverse primers that were extended with HindIII and BamHI restrictions sites. The amplified PCR products were digested and cloned to pT7_3v5 plasmid containing a gene for puromycin selection. The AAC or SCS DKO cells were transfected with the NotI-linearized plasmid. Addback cell lines were grown in the presence of G418, hygromycin, and puromycin, and the expression of the ectopic alleles was initiated by the addition of 1μg/ml tetracycline.

SCS conditional DKO (cDKO) cell line was generated using SCS single KO cell line which was transfected with pT7_3v5_SCS linearized plasmid. After successful selection with puromycin, the second allele was replaced using the pLEW90_SCS_5´/ 3´UTRs construct. The transfection and selection were done in the presence of tetracycline ensuring expression of regulatable SCS. SCS cDKO was grown in the presence of tetracycline, G418, hygromycin, and phleomycin. Suppression of the ectopic allele expression was done by washing the cells twice in tetracycline-free media.

### Measurement of ΔΨ_m_ using flow cytometry

The ΔΨ_m_ was determined utilizing the red-fluorescent dye tetramethylrhodamine ethyl ester (TMRE, Invitrogen). Cells were grown in log-phase for a few days prior the experiment. In a specific case, the cells were pre-treated with oligomycin at the sublethal concentration of 250 μg/ml for 24 hours before the experiment. Then, in total, 5 × 10^6^ of oligomycin treated or untreated cells were pelleted (1,300 g, 10 min, room temperature), resuspended in 1 ml of the appropriate medium, incubated with 60 nM TMRE for 30 min at 37°C, washed in PBS, resuspended in PBS-G (PBS, 6 mM glucose) and immediately analyzed by flow cytometry (BD FACS Canto II Instrument). In the case of oligomycin-treated cells, the 250 μg/ml of oligomycin was maintained in all buffers and washes. For each sample, 10,000 fluorescent events were collected. Treatment with the protonophore FCCP (20 μM) for 10 min was used as a control for mitochondrial membrane depolarization. Data were evaluated using BD FACSDiva (BD Company) software.

### SDS PAGE, Western blots, antibody production

Cell cultures were harvested at 1,300 g at 4°C for 10 minutes, washed with 1x PBS and the lysates were prepared at concentration 1 x 10^7^ cells/30 μl using 1 x PBS, 6% sodium dodecyl sulfate, 300 mM DTT, 150 mM Tris HCl, 30% glycerol, and 0.02% Bromophenol Blue. Samples were boiled for 7 minutes at 97°C and stored at -20°C. Proteins were resolved on SDS-PAGE gels (BioRad 4568093, Invitrogen XP04202BOX) using 10^7^ cells/sample. Proteins were blotted onto PVDF membrane (Thermo Scientific) and probed with corresponding monoclonal (mAb) or polyclonal (pAb) antibodies. This was followed by probing with secondary HRP conjugated anti-mouse or anti-rabbit antibody (1:2,000 dilution, SIGMA). Proteins were visualized using the Clarity Western ECL substrate (Bio-Rad 1705060EM) on a ChemiDoc instrument (Bio-Rad). The PageRuler pre-stained protein standard (Fermentas) was used to determine the size of the detected bands. AAC and SCS pAb were prepared for the purpose of this study. Open reading frames of AAC and SCS beta subunit were cloned in *E*. *coli* expression plasmid pSKB3. Proteins were overexpressed in *E*. *coli* BL21 cells, solubilized by sarkosyl, and purified by high-performance liquid chromatography. Antigens were sent to David’s Biotechnologie (Germany) for pAb production. Primary antibodies used in this paper are following: pAb anti-AAC (1:1,000, 34 kDa), pAb anti-SCS (1:1,000, 45 kDa), pAb anti-APRT (1:500, 26 kDa), pAb anti-p18 (1:1,000, 18 kDa) and mAb anti-HSP70 (1,5,000, 72 kDa).

### Digitonin subcellular fractionation

Whole cell lysates (WCL) were prepared from BSF 427 for SCS localization and cell lines expressing mitochondrial (mito) or cytosolic (cyto) luciferase. For the digitonin fractionation, 1x10^8^ cells were harvested and washed with 1x PBS-G. Pellet was resuspended in 500 μl of SoTe (0.6 M Sorbitol, 2 mM EDTA, 20 mM Tris-HCl pH 7.5) and lysed with 500 μl of SoTe with 0.03% digitonin. Samples were incubated on ice for 5 minutes and centrifuged at 7,000 rpm for 3 minutes at 4°C. The supernatant was harvested as a cytosolic fraction and the pellet was resuspended in 1x PBS as a mitochondrial fraction. WCL and the fractions were resolved by SDS-PAGE.

### Measurement of ΔΨ_m_ using Safranin O dye

This method was performed as described previously [22]. Briefly, the in situ ΔΨ_m_ was measured using Safranin O dye (Sigma, S2255-25G). 2 x 10^7^ cells were centrifuged for 10 minutes at room temperature at 1,300 g and washed with ANT buffer containing 8 mM KCl, 110 mM K-gluconate, 10 mM Mannitol, 10 mM NaCl, 10 mM free acid HEPES, 10 mM K_2_HPO_4_, 0.015 mM EGTA potassium salt, 0.5 mg/ml fatty acid free bovine serum albumin, and 1.5 mM MgCl_2_ at pH 7.25. The cell pellet was resuspended with ANT buffer with 4 μM digitonin and 5 μM Safranin O. Fluorescence was recorded in a Hitachi F-7100 spectrofluorometer (Hitachi High Technologies) at a 5-Hz acquisition rate, using 495 nm excitation and 585 nm emission wavelengths. Samples were measured at room temperature and stirred during the experiment. Where indicated, 1 mM ATP as a substrate and inhibitors (1 μM CATR or 10 μM oligomycin) were added. Finally, the SF6847 uncoupler was used as a control of the maximal depolarization.

### SCS activity assay

The organellar pellet from 5 x 10^8^ digitonin-fractionated cells was resuspended in ANT buffer, sonicated 3 times for 10 seconds at 20% power. The sample was spun down at 16,000 g for 5 min and supernatant containing mitochondrial matrix was subjected to SCS activity assay. The activity was assayed in ANT buffer in the presence of succinyl-CoA (0.2 mM), ADP (2 mM), Ellman´s reagent (5,5’-dithio-bis-[2-nitrobenzoic acid], DNTB (0.2 mM)) at 30°C. The released CoA-SH reacted with DNTB forming thio-nitrobenzoate anion (TNB) which production in time was monitored spectrophotometrically at 412 nM using a Tecan Infinite M200 plate reader. One unit is defined as an enzyme activity that converts one nanomole of succinyl CoA to CoA-SH in 1 minute per 1 mg of total protein.

### Alamar blue-based cell viability assay

Corresponding *T*. *brucei* cell lines were plated in transparent 96-well plates in a concentration of 5 x 10^3^ cells/ml in 200 μl per well. Cells were grown in the presence of different CATR concentrations between 1 μM to 500 μM or in the presence of TPMP between 0.3 nM to 500 μM for 72 hours in standard cultivation conditions. After 72 hours 20 μl of 125 μg/ml of resazurin (Sigma, R7017-1G) was added to each well. After 24 hours the fluorescence was measured using Tecan Spark set up for 544 nm wavelength for excitation and 590 nm for emission. Data were analyzed using GraphPad Prism 9 to establish the EC_50_ values.

### *In vivo* ATP measurements

5 x 10^6^ cells with constitutively expressed luciferase in cytosol or mitochondrion were centrifuged at 1,300 g for 7 minutes at room temperature. Cells were washed with 1x PBS and resuspended in 160 μl of HEPES-LUC buffer containing 20 mM HEPES, 116 mM NaCl, 5.6 mM KCl, 8 mM MgSO_4_ and 1.8 mM CaCl_2_ at pH 7.4. Cells were immediately placed in white bioluminescence 96 well plates, the background luminescence was measured by the Tecan Spark and 40 μl of 250 μM luciferin was injected in each sample. The luminescence was measured for 20 cycles and where indicated 10 mM glucose was injected and changes of luminescence were recorded for another 35 cycles.

### Animal experiments

Groups of 7 mice were used for each of the cell lines. Mice were infected by 10^5^ cells via 100 μl intraperitoneal injection of either BSF 427, AAC DKO, AAC DKO addback, SCS DKO, and SCS DKO addback. Mice injected with tetracycline induced addback cell lines were put on doxycycline (200 μg/ml doxycycline and 5% sucrose) drinking regime 24 hours before injection. Blood samples from a tail prick were diluted in 1x SSC and 3.7% formaldehyde, and the parasitemia levels were counted using a hemocytometer (Counting Chamber CE NeubauerIMP DL). Parasitemia counts were observed for 15 days and mice displaying impaired health or a parasite load over 10^8^ cells/ml of blood were euthanized.

### NMR experiments

BSF 427, AAC DKO and SCS DKO trypanosomes were grown in log-phase in HMI11 media supplemented with the respective drugs. 10^7^ cells were collected by centrifugation at 1,400 g for 10 minutes at RT and washed with incubation buffer (PBS buffer supplemented with 5 g/L NaHCO_3_, pH 7.4) with the addition of 1 mM of the respective carbon source. Next, the cells were incubated in preheated plates until the cells manage to keep cell integrity (validated by microscopic observation, appr. 2.5 hours) at 37°C with incubation buffer containing uniformly labeled [U-^13^C]-glucose (4 mM) in the presence or absence of the 4 mM amino acid threonine in a total volume of 1 ml. The same experiment was carried out with ordinary ^12^C glucose as the only carbon source. Following centrifugation at 8,000 g for 1 minute at RT, the supernatant was collected and a proton NMR (^1^H-NMR) spectra analysis was performed as described in [31].

### LC-MS metabolomics

5 x 10^7^ cells for each sample were rapidly cooled down in an ethanol-dry ice bath, centrifuged at 1,300 g for 10 minutes at 4°C, and washed with 1x PBS. Pellet was resuspended in 100 μl of extraction solvent containing chloroform, methanol, and water (1:3:1 volume ratio). Samples were shaken for 1 hour at 4°C, pelleted at 13,000 g for 10 minutes at 4°C and the supernatants were stored at -80°C until analysis. The used metabolomic methods were described in detail elsewhere [63,64]. Briefly, an Orbitrap Q Exactive Plus mass spectrometer coupled to an LC Dionex Ultimate 3000 (all Thermo Fisher Scientific, San Jose, CA, USA) was used for metabolite profiling. LC condition: column SeQuant ZIC-pHILIC 150 mm x 4.6 mm i.d., 5 μm, (Merck KGaA, Darmstadt, Germany); flow rate of 450 μl/min; injection volume of 5 μl; column temperature of 35°C; mobile phase A = acetonitrile and B = 20 mmol/l aqueous ammonium carbonate (pH 9.2; adjusted with NH_4_OH); gradient: 0 min, 20% B; 20 min, 80% B; 20.1 min, 95% B; 23.3 min, 95% B; 23.4 min, 20% B; 30.0 min 20% B. The Q-Exactive settings were: mass range 70–1050 Daltons; 70,000 resolution; electrospray ion source operated in the positive and negative modes. Data were processed using Xcalibur software, version 4.0 (Thermo Fisher Scientific, San Jose, CA, USA), and an in-house developed Metabolite Mapper platform containing more than 1,500 metabolites manually annotated against authentic standards. Metabolomic data are available at MetaboLights database [65] with identifier MTBLS8490 (www.ebi.ac.uk/metabolights/MTBLS8490).

### Mass spectrometry sample preparation, MS measurement, and proteomics data analysis

*T*. *brucei* BSF 427, SCS and AAC DKO cells (10^8^ cells/replicate) were washed three times in 10 ml of phosphate-buffered saline (PBS) and lysed in 6% sodium dodecyl sulfate (SDS), 300 mM DTT, and 150 mM Tris-HCl (pH 6.8), 30% glycerol, and 0.02% Bromophenol Blue. Samples were loadedon a NOVEX NuPage 4%-12% gradient gel (Thermo Fisher Scientific, Waltham, MA), run for 10 minutes at 180 V, and stained with Coommassie G250 (Roth, Karlsruhe, Germany). Each lane was cut and the minced gel pieces were transferred to an Eppendorf tube for destaining with 50% ethanol/50 mM ABC buffer pH 8.0. The gel pieces were dried and subsequently reduced (10 mM DTT/50 mM ABC buffer pH 8.0), alkylated (55 mM iodoacetamide/50 mM ABC buffer pH 8.0), and digested with 1 μg trypsin overnight at 37°C. The tryptic peptides were eluted from the gel pieces with pure acetonitrile and a mixture of 30% acetonitrile in 50 mM ABC pH 8.0. The acetonitrile was evaporated in a concentrator (Eppendorf, Germany) and the peptides stored on a StageTip [66]. The proteomic measurement was performed on an Exactive 480 mass spectrometer (Thermo Fisher Scientific, Bremen, Germany) with an online-mounted C18-packed capillary column (New Objective, Woburn, MA) by eluting along a 90-minute gradient of 2% to 40% acetonitrile using an EasyLC 1200 uHPLC system (Thermo Fisher Scientific, Bremen, Germany). The mass spectrometer was operated with a top20 data-dependent acquisition (DDA) mode. Data analysis was performed in MaxQuant version 1.5.2.8 using the tritrypDB-8.1 TbruceiTREU927_AnnotatedProteins database (11,567 entries) and standard settings, except activating the match between run feature and the label-free quantification (LFQ) algorithm. Protein groups marked as contaminants, reverse entries, and only identified by site were removed prior to bioinformatics analysis, as well as protein groups with less than 2 peptides (minimum 1 unique). Additional information like gene names and descriptions were extracted from the fasta header and attached to the individual protein groups. The mass spectrometry proteomics data have been deposited to the ProteomeXchange Consortium via the PRIDE [67] partner repository with the dataset identifier PXD044938.

### Statistical analysis

The number of replicates, controls, and statistical tests are in accordance with published studies employing comparable techniques and are generally accepted in the field. Statistical differences were analyzed with Prism software (version 8.2.1, GraphPad software). Comparisons of two groups were calculated with two-tailed paired *t* test. A *P* value of less than 0.05 was considered statistically significant. Quantitative mass spectrometry experiments were performed in four biological replicates.

## Supporting information

S1 Fig Proteomic and metabolomic profiling of AAC DKO cells. (A) Volcano plots showing a comparison of protein expression levels (3654 protein groups) between BSF 427 and AAC DKO cells. Log2 fold change values of averaged LFQ intensities from quadruplicate experiments are plotted against the respective −log10-transformed *P* values. Significantly changed hypothetical proteins are shown in blue, down-regulated cytosolic ribosomal proteins are shown in dark red. Mitochondrial enzymes involved in amino and keto acid oxidation including TCA cycle enzymes are highlighted in orange. ME, malic enzyme; IDH, isocitrate dehydrogenase; SDH1, succinate dehydrogenase subunit 1; AAT, alanine aminotransferase; PDH E3, subunit of pyruvate dehydrogenase; KDH E2, subunit of α-ketoglutarate dehydrogenase; BCKD E2, subunit of branch chain keto acid dehydrogenase; MPC2, mitochondrial pyruvate carrier 2; MCP14, mitochondrial carrier protein 14. (B) Volcano plot showing the detected metabolites (124 metabolites) analyzed in BSF 427 and AAC DKO cells. Log2 fold change values of the average of mean peak area from quadruplicate experiments are plotted against the respective −log10 transformed *P* values. AAs, amino acids.
(TIF)

S2 Fig Proteomic and metabolomic profiling of SCS DKO cells. (A) Volcano plots showing a comparison of protein expression levels (3,654 protein groups) between BSF 427 and SCS DKO cells. Log2 fold change values of averaged LFQ intensities from quadruplicate experiments are plotted against the respective −log10-transformed *P* values. Significantly changed hypothetical proteins are shown in blue. SCS sub α, subunit α of SCS α/β complex. (B) Volcano plot showing the detected metabolites (125 metabolites) analyzed in BSF 427 and AAC DKO cells. Log2 fold change values of the average of mean peak area from quadruplicate experiments are plotted against the respective −log10 transformed *P* values. Metabolites derived from the reaction of TCA cycle, glutamine/glutamate metabolism, serin/threonine/alanine/aspartate metabolism, pentose phosphate pathway and oxidative stress are highlighted in red, green, purple, blue and yellow respectively. α-KG, α-ketoglutarate.
(TIF)

S1 Table Proteomic analysis of AAC DKO and SCS DKO cells. Sheet 1 contains Tb927 gene IDs and description for 3,654 protein groups identified by a minimum of 2 peptides (1 unique) and present in at least two out of four replications. Sheet 2 contains protein groups identified in BSF 427 cells and compared to AAC DKO. Sheet 3 contains protein groups differentially expressed (log2 fold change < - 0.4, log2 fold change > 0.5) which passed threshold of *p-*value of 0.05. Sheet 4 contains protein groups identified in BSF 427 cells and compared to SCS DKO. Sheet 5 contains protein groups differentially expressed (log2 fold change < - 0.4, log2 fold change > 0.5) which passed threshold of *p-*value of 0.05.
(XLSX)

S2 Table Metabolomic analysis of AAC DKO cells. LC-MS metabolomic data.
(XLSX)

S3 Table Metabolomic analysis of SCS DKO cells. LC-MS metabolomic data.
(XLSX)

S4 Table Excreted end-products from metabolism of glucose and threonine in BSF trypanosomes. Parasites were incubated with 4 mM glucose or with [U-_13_C]-glucose with or without 4 mM threonine. ICS (internal carbon source): intracellular carbon source of unknown origin metabolized by the BSF trypanosomes. Amounts of end-products excreted (here malate) from the carbon source indicated in brackets, expressed as nmoles excreted per h and per 108 cells. *nd*: not detectable.
(XLSX)

S5 Table List of oligonucleotides used in the study. (XLSX)
