# Supplementary figures and images for: Mitochondrion of the Trypanosoma brucei long slender bloodstream form is capable of ATP production by substrate-level phosphorylation
Source: PLoS Pathog. 2023 Oct 11;19(10):e1011699. doi: 10.1371/journal.ppat.1011699 (PMC10593219; doi:10.1371/journal.ppat.1011699)

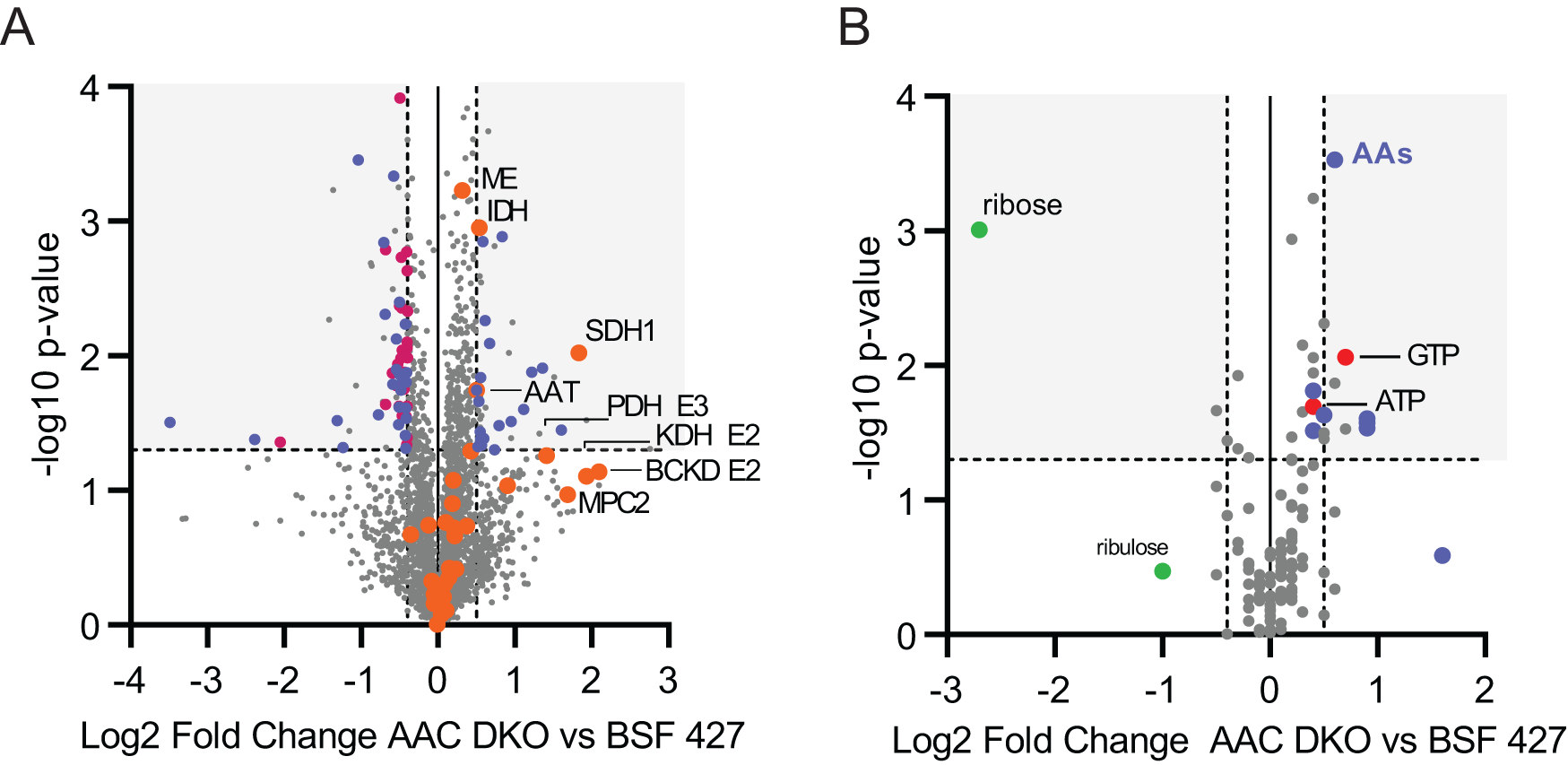

Supplement: S1 Fig — (A) Volcano plots showing a comparison of protein expression levels (3654 protein groups) between BSF 427 and AAC DKO cells. Log2 fold change values of averaged LFQ intensities from quadruplicate experiments are plotted against the respective −log10-transformed P values. Significantly changed hypothetical proteins are shown in blue, down-regulated cytosolic ribosomal proteins are shown in dark red. Mitochondrial enzymes involved in amino and keto acid oxidation including TCA cycle enzymes are highlighted in orange. ME, malic enzyme; IDH, isocitrate dehydrogenase; SDH1, succinate dehydrogenase subunit 1; AAT, alanine aminotransferase; PDH E3, subunit of pyruvate dehydrogenase; KDH E2, subunit of α-ketoglutarate dehydrogenase; BCKD E2, subunit of branch chain keto acid dehydrogenase; MPC2, mitochondrial pyruvate carrier 2; MCP14, mitochondrial carrier protein 14. (B) Volcano plot showing the detected metabolites (124 metabolites) analyzed in BSF 427 and AAC DKO cells. Log2 fold change values of the average of mean peak area from quadruplicate experiments are plotted against the respective −log10 transformed P values. AAs, amino acids. (TIF) [file ppat.1011699.s001.tif]

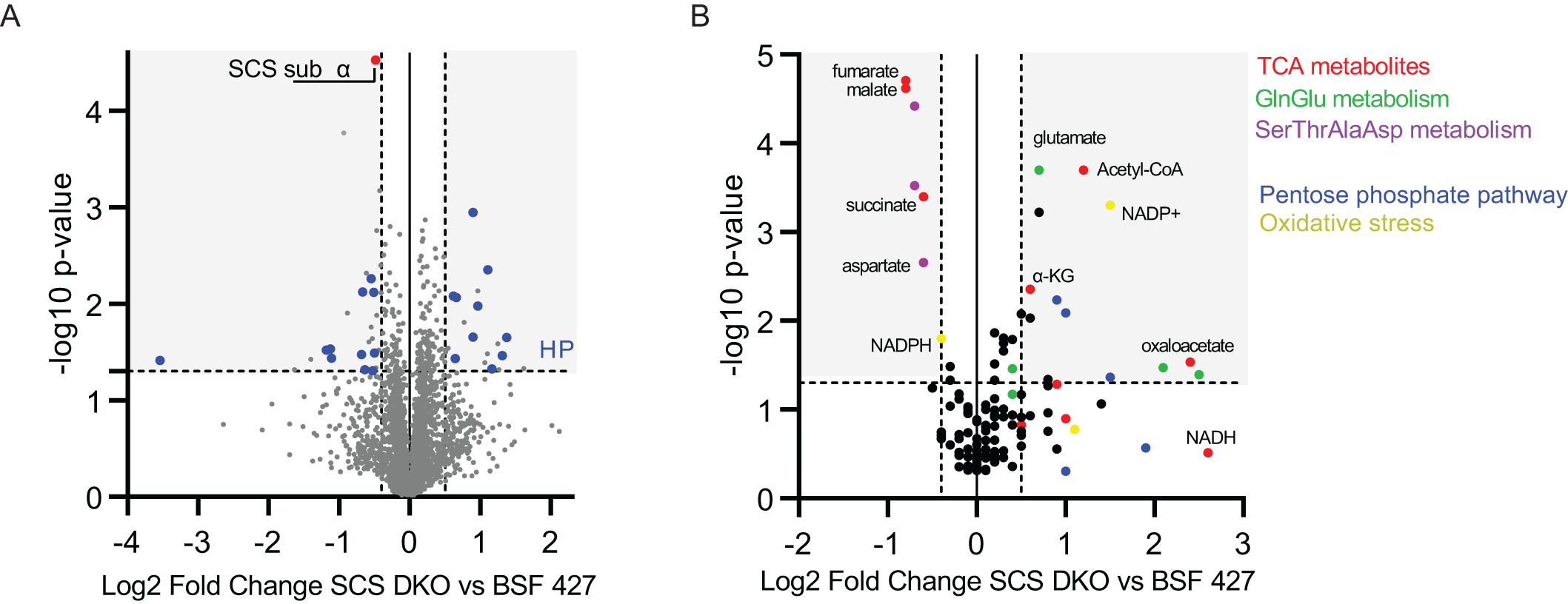

Supplement: S2 Fig — (A) Volcano plots showing a comparison of protein expression levels (3,654 protein groups) between BSF 427 and SCS DKO cells. Log2 fold change values of averaged LFQ intensities from quadruplicate experiments are plotted against the respective −log10-transformed P values. Significantly changed hypothetical proteins are shown in blue. SCS sub α, subunit α of SCS α/β complex. (B) Volcano plot showing the detected metabolites (125 metabolites) analyzed in BSF 427 and AAC DKO cells. Log2 fold change values of the average of mean peak area from quadruplicate experiments are plotted against the respective −log10 transformed P values. Metabolites derived from the reaction of TCA cycle, glutamine/glutamate metabolism, serin/threonine/alanine/aspartate metabolism, pentose phosphate pathway and oxidative stress are highlighted in red, green, purple, blue and yellow respectively. α-KG, α-ketoglutarate. (TIF) [file ppat.1011699.s002.tif]
