# Supplementary material for: Mitochondrion of the Trypanosoma brucei long slender bloodstream form is capable of ATP production by substrate-level phosphorylation
Source: PLoS Pathog. 2023 Oct 11;19(10):e1011699. doi: 10.1371/journal.ppat.1011699 (PMC10593219; doi:10.1371/journal.ppat.1011699)
Supplement: Attachment — Submitted filename: Response to the reviewers_Taleva et al. FIN.docx [file ppat.1011699.s008.docx]

*Sept 12^th^, 2023*

*Dear Editor, dear Laurie,*

*Thank you very much for your feedback on our manuscript. We also thank both the reviewers for taking time out of their busy schedule to provide us with fair criticism and recommendations that we believe have helped us to improve the quality of the manuscript.*

*We agree with the reviewer 2 that the title of the manuscript as well as the text might mislead the reader into thinking that our data also refer to the cell-cycle arrested transmission form of the parasite, and at his request we have modified the title and also tried in many places in the text to indicate that we have worked with a proliferating culture of the long slender monomorphic trypanosomes. At his request, we have also moved the original figures 4 and 7 to the Supplementary Data and shortened the discussion.*

*We hope that the improved manuscript is now acceptable for publication in PLoS Pathogens.*

*Sincerely,*

*Alena*

**Part I - Summary**

 Reviewer #1: Previously, ATP production in bloodstream-form (BSF) Trypanosoma brucei was considered to rely entirely on glycolysis. The parasite’s single mitochondrion is highly repressed in the BSF. Energy required for a few remaining essential mitochondrial functions, such as the generation of a transmembrane proton-motive force (pmf) for import of solutes and proteins, was supposed to come from glycolytically produced ATP imported from the cytosol. The F1FO-ATPase would then act in reverse to produce the pmf. Data to support this notion have previously been reported.

However, more recent experiments provided indications for some additional mitochondrial functions in the BSF that could provide ATP by substrate-level phosphorylation. In this manuscript, Alena Zikova and her collaborators describe how they analyzed – and confirmed the existence of – the possible routes by which the BSF mitochondrion acquires its ATP: by import from the cytosol as well as different substrate-level phosphorylation reactions within the organelle. They discuss how the relative contributions of each of the different routes may be dependent on various situations.

The scientific approach of the research has been very systematic, and appropriate methods of molecular biology, biochemistry and -omics (proteomics, metabolomics) have been used to address the research questions. The experiments have been well performed and yielded results that allowed drawing convincing conclusions. Statistical analysis of data was – according to my judgement – appropriately done when necessary. The work and methodologies have been well described, and the manuscript contains an interesting, highly relevant Discussion section.

I have only minor comments and questions, specified below.

*We thank the reviewer for a positive feedback on our study.*

Reviewer #2: Strength: Authors in this manuscript showed that the bloodstream form of Trypanosoma brucei produces ATP in mitochondria by substrate level phosphorylation to run ATP-synthase in reverse orientation for generation of mitochondrial membrane potential. They also provide evidence that mitochondrial ADP/ATP carrier (AAC) is the only source to transport ATP from cytosol to mitochondria; however, AAC is dispensable for the survival of the bloodstream form. Overall experiments are well designed, and results are conclusive.

Weakness: Authors used monomorphic bloodstream form culture for their studies. However, they generalized their conclusion for all sorts of BF particularly in the title and throughout the manuscript. This is somewhat misleading. There are previous reports that the stumpy BF utilizes 2-oxoglutarate and produces ATP in the mitochondria [Bienen et al., 1993, Eur. J. Biochem. 216,75], which was not mentioned. Authors need to modify the title to highlight the novelty of their findings.

*We understand the reviewer's reservation and have accordingly made it clear at the beginning of the manuscript and in many different places that we have worked with proliferative long slender monomorphic parasites. We also added a sentence referring to the fact that stumpy form parasites are able to respire on a-ketoglutarate and possibly produce ATP by substrate phosphorylation. The reference to Bienen et al, 1993 has been added.*

**Part II – Major Issues: Key Experiments Required for Acceptance**

Reviewer #1: I consider no need for new experiments or modifications of existing experiments

Reviewer #2: No additional experiments are required.

Authors only used monomorphic, cultured bloodstream form cell lines. Therefore, the results obtained from these studies shouldn't be generalized for all bloodstream forms.

Title is misleading. It has been reported previously that the bloodstream form can produce ATP by substrate level phosphorylation.

*As explained above, we agree with the reviewer and have adjusted the title and text accordingly.*

**Part III – Minor Issues: Editorial and Data Presentation Modifications**

**Reviewer #1:** Some minor scientific issues:

1. Line 102: It seems appropriate to make a reference to Van Hellemond et al, PNAS 1998, who – still in the pro-genomics era, when studying acetate production in trypanosomatids – made the surprising finding of the ASCT/SCS cycle in trypanosomatids (although not in BSF T. brucei, but in procyclic T. brucei, Leishmania and Phytomonas). Until then (and still often), these enzymes were considered typical for anaerobic organisms.

*We appreciate the reviewer's insight as we should not overlook the original discoveries made long ago. The reference was added*.

2. Lines 133-134: I doubt if CMM better represents the in vivo situation than HMI medium. It may not be true with respect to amino acids. Probably it would be better to write 'which, with its 10 mM glucose, represents better – although still well above – the extracellular glucose concentration in the mammalian host’ (i.e., about 5 mM in human blood; 2-4 mM in ruminants). However, how relevant is it anyway, with glucose transport known to control the glycolytic flux (data from papers by Bakker and Haanstra), rendering it very unlikely that the intracellular glucose concentration is affected by changes in the external one at concentrations well above the Km of about 0.5-1 mM of the facilitated-diffusion transporter involved?

*Again, the reviewer is correct and we have changed the statement accordingly (lines 163-164).*

3. Lines 391-392: The information in this sentence is not accurately formulated, because it has been well known for years that metabolism of other substrates than glucose also contribute as carbon (i.e., for producing biomass) to T. brucei, such as amino acids and fatty acids. Glucose was considered the exclusive energy source, although in the presence of glycerol trypanosomes could be kept alive (by providing ATP) for some time without enabling them to proliferate. The research described in references 47 and 48 showed that, in the absence of glucose, glycerol can be used as both energy and carbon source. The novel finding of these references was that BSF can produce higher-molecular weight carbons from glycerol by gluconeogenesis, enabling cell proliferation.

*Yes. We have corrected the text (line 520).*

4. Line 425: The suggestion that evasion of the immune response costs greater quantities of cytosolic ATP is unlikely. The major mechanism for evasion of the immune response is due to recycling the VSG coat, eliminating attached antibodies, driven by the trypanosome’s motility caused by flagellar beating. These processes also occur in vitro. Moreover, very recently published calculations show that this process (flagellar beating /motility /VSG recycling) involves only a very minor part of the BSF ATP budget (Nascimento et al., PLoS Pathog. 19(7): e1011522).

*We changed the text accordinaly (line 565) and we also refer to the Nascimento et al., study in text.*

5. Lines 515-516: The (predominantly) cytosolic localization of alanine aminotransferase in BSF trypanosomes is not only ‘thought’ but has been clearly demonstrated by cell fractionation of trypanosomes, as reported in various publications. See for example Steiger et al., Eur. J. Biochem. 105, 1980, 163-175.

*The text was changed accordingly and the reference was added.*

Textual and other minor issues:

1. Lines 4-5: Some authors are listed with their given name followed by their surname, while for other authors the surname is followed by the given name.

2. Line 126: Change ‘homology recombination’ to ‘homologous recombination’.

3. Line 136: Change 'was' to 'were'.

4. Line 176: At this first mentioning of the v5-tag (or in the legend of Fig. 3B), explain for non-informed readers the purpose of the tag (i.e., a peptide for immuno detection).

5. Lines 187 and 403: Change ‘mitochondria’ to ‘mitochondrion’.

6. Line 268-269: Change 'are' to 'is' and 'activity' to 'activities' (or change only 'were' in line 269 to 'was').

7. Line 302: 'downregulation' seems in this context a more appropriate word than 'impairment'.

8. Line 306: Alterations in TCA cycle metabolites were already mentioned above in lines 298-301.

9. Line 311: Add also ‘AAC DKO cells’ (they are shown in Fig. 8B, but this panel should be presented as 8C).

10. Line 318: Figure 8B should be 8C. Figure 8B (about AAC DKO cells) is not discussed in this section.

11. Line 347: 'intracellular'? Should it not be 'intramitochondrial'?

12. Line 429: Add 'in' to give: ‘when in in vivo or CMM-gly culture conditions'.

13. Line 431: 'Finally, we can conclude'? I suppose the authors meant something as: 'From all data together we can now conclude'.

14. Line 434: Change ‘occurs’ to 'was considered to occur'.

15. Lines 484-491: The text refers to Fig. 12A and 12C (and later – in line 549 - to 12D). No reference to panel B is made. I suggest adding such reference somewhere (because the panel is useful in the context of the other panels, and the journal would probably have all panels mentioned in the text).

16. Line 496: Correct to '10-times'.

17. Line 555: Add 'across the mitochondrial inner membrane' at the end of the sentence'.

18. Line 584: 'repressor under 10% T7 RNAP promoter'? The meaning of 10% is not clear to this reviewer.

19. Lines 588, 595, 606 and 619: The cassette/plasmid is not transfected but cells are transfected with the cassette/plasmid.

20. Line 594: Change 'inserted to' to 'inserted in’ (or into).

21. Line 625: Change 'was' to 'were'.

22. Line 632: Change 'with the sublethal concentration' to 'at the sublethal concentration.'

23. Lines 653 and 654: ‘coli’ with lower case c.

24. Line 721: Correct: °C (no superscript 0 but °) and labeled.

25. Line 785: Change AAC DKO cells to SCoAS DKO cells.

26. Lines 786 and 870: Delete 'the'.

27. Lines 805 and 882: Delete 'resistance' in front of ‘genes’ (it is redundant with the 2nd 'resistance' – linked to the antibiotic - later in the sentence).

28. Line 845: Add an ‘l’ to give ‘panel’.

29. Lines 849-850: Change 'levels bioluminescence' to 'bioluminescence levels'.

30. Line 918: (1) The panels of Figure 12 remain to be labeled A, B and C. (2) The panels are shown in the order (A) BSF427, (C) AAC DKO and (B) SCS DKO, not A, B and C.

31. Lines 945-946: The sentence is difficult to understand. Better (?): 'Data shown in the bars are derived from experiments of which representative graphs are shown in panel D.'

32. Figures 3D and F: Correct ‘luminiscence’ to ‘luminescence’.

*All minor issues have been corrected. We would like to thank the reviewer for this impressive and thorough work.*

**Reviewer #2:** Too many figures. Some proteomics and metabolomics data, like Fig. 4A & B, and 7A & B, could be presented in the supplementary material. As stated by the authors these analyses couldn’t detect any major metabolic changes.

*As suggested by the reviewer, we moved the Figures 4 and 7 to the supplementary material.*

Fig. 6E, SCS activity appears reduced in AAC DKO cells, although it was considered by the authors as non-significant. What is the basis for this consideration? How this reduction could be explained?

*It is difficult to speculate on the reduced SCS activity in AAC DKO, although the opposite would be expected. Because the difference is not statistically significant, we do not venture to speculate and prefer to conclude that there are no major changes in SCS activity between BSF 427 and AAC DKO cells*.

Discussion is too long, which needs to be shortened. Authors could focus on the novelty of their finding as discussed in the beginning of this section. It is also necessary to mention that studies were performed using the monomorphic BF. There are possibilities that DKO of AAC and SCS may generate slightly altered phenotypes than that observed here.

*As suggested by the reviewer, we have tried to consolidate the discussion, although reviewer 1 found it very good. Nevertheless, we have deleted two entire paragraphs, the information from which has been inserted in a more consolidated version in the results section. We hope this is satisfactory to the reviewer.*
