# Supplementary material for: Mitochondrion of the Trypanosoma brucei long slender bloodstream form is capable of ATP production by substrate-level phosphorylation
Source: PLoS Pathog. 2023 Oct 11;19(10):e1011699. doi: 10.1371/journal.ppat.1011699 (PMC10593219; doi:10.1371/journal.ppat.1011699)
Supplement: Peer review history

# Peer review: Decision Letter 0

**DOI:** 10.1371/journal.ppat.1011699.r001

**Authors:** Laurie Read, James J Collins III

7 Aug 2023

Dear Alena,

Thank you very much for submitting your manuscript "Trypanosoma brucei bloodstream form mitochondrion is capable of ATP production by substrate-level phoshorylation" for consideration at PLOS Pathogens. As with all papers reviewed by the journal, your manuscript was reviewed by members of the editorial board and by several independent reviewers. The reviewers appreciated the attention to an important topic but identified some aspects of the manuscript that should be improved.  While no new experiments are needed, we ask you to modify the manuscript according to the review recommendations before we can consider your manuscript for acceptance. Your revisions should address the specific points made by each reviewer. Note that Reviewer 2 feels the title of your manuscript is misleading in suggesting that this is the first report of ATP production in bloodstream mitochondria given the 1993 Beinen article and data in reference 35 of your manuscript; they suggest you use a more specific title.  Please give this due consideration.

Please prepare and submit your revised manuscript within 30 days. If you anticipate any delay, please let us know the expected resubmission date by replying to this email.

When you are ready to resubmit, please upload the following:

[1] A letter containing a detailed list of your responses to all review comments, and a description of the changes you have made in the manuscript.

Please note while forming your response, if your article is accepted, you may have the opportunity to make the peer review history publicly available. The record will include editor decision letters (with reviews) and your responses to reviewer comments. If eligible, we will contact you to opt in or out

[2] Two versions of the revised manuscript: one with either highlights or tracked changes denoting where the text has been changed; the other a clean version (uploaded as the manuscript file).

Important additional instructions are given below your reviewer comments.

Thank you again for your submission to our journal. We hope that our editorial process has been constructive so far, and we welcome your feedback at any time. Please don't hesitate to contact us if you have any questions or comments.

Sincerely,

Laurie Read

Pearls Editor

PLOS Pathogens

James Collins III

Section Editor

PLOS Pathogens

Kasturi Haldar

Editor-in-Chief

PLOS Pathogens

​orcid.org/0000-0001-5065-158X

Michael Malim

Editor-in-Chief

PLOS Pathogens

orcid.org/0000-0002-7699-2064

***********************

Reviewer Comments (if any, and for reference):

Reviewer's Responses to Questions

**Part I - Summary**

Please use this section to discuss strengths/weaknesses of study, novelty/significance, general execution and scholarship.

Reviewer #1: Previously, ATP production in bloodstream-form (BSF) Trypanosoma brucei was considered to rely entirely on glycolysis. The parasite’s single mitochondrion is highly repressed in the BSF. Energy required for a few remaining essential mitochondrial functions, such as the generation of a transmembrane proton-motive force (pmf) for import of solutes and proteins, was supposed to come from glycolytically produced ATP imported from the cytosol. The F1FO-ATPase would then act in reverse to produce the pmf. Data to support this notion have previously been reported.

However, more recent experiments provided indications for some additional mitochondrial functions in the BSF that could provide ATP by substrate-level phosphorylation. In this manuscript, Alena Zikova and her collaborators describe how they analyzed – and confirmed the existence of – the possible routes by which the BSF mitochondrion acquires its ATP: by import from the cytosol as well as different substrate-level phosphorylation reactions within the organelle. They discuss how the relative contributions of each of the different routes may be dependent on various situations.

The scientific approach of the research has been very systematic, and appropriate methods of molecular biology, biochemistry and -omics (proteomics, metabolomics) have been used to address the research questions. The experiments have been well performed and yielded results that allowed drawing convincing conclusions. Statistical analysis of data was – according to my judgement – appropriately done when necessary. The work and methodologies have been well described, and the manuscript contains an interesting, highly relevant Discussion section.

I have only minor comments and questions, specified below.

Reviewer #2: Strength: Authors in this manuscript showed that the bloodstream form of Trypanosoma brucei produces ATP in mitochondria by substrate level phosphorylation to run ATP-synthase in reverse orientation for generation of mitochondrial membrane potential. They also provide evidence that mitochondrial ADP/ATP carrier (AAC) is the only source to transport ATP from cytosol to mitochondria; however, AAC is dispensable for the survival of the bloodstream form. Overall experiments are well designed, and results are conclusive.

Weakness: Authors used monomorphic bloodstream form culture for their studies. However, they generalized their conclusion for all sorts of BF particularly in the title and throughout the manuscript. This is somewhat misleading. There are previous reports that the stumpy BF utilizes 2-oxoglutarate and produces ATP in the mitochondria [Bienen et al., 1993, Eur. J. Biochem. 216,75], which was not mentioned. Authors need to modify the title to highlight the novelty of their findings.

**********

**Part II – Major Issues: Key Experiments Required for Acceptance**

Please use this section to detail the key new experiments or modifications of existing experiments that should be absolutely required to validate study conclusions.

Generally, there should be no more than 3 such required experiments or major modifications for a "Major Revision" recommendation. If more than 3 experiments are necessary to validate the study conclusions, then you are encouraged to recommend "Reject".

Reviewer #1: I consider no need for new experiments or modifications of existing experiments

Reviewer #2: No additional experiments are required.

Authors only used monomorphic, cultured bloodstream form cell lines. Therefore, the results obtained from these studies shouldn't be generalized for all bloodstream forms.

Title is misleading. It has been reported previously that the bloodstream form can produce ATP by substrate level phosphorylation.

**********

**Part III – Minor Issues: Editorial and Data Presentation Modifications**

Please use this section for editorial suggestions as well as relatively minor modifications of existing data that would enhance clarity.

Reviewer #1: Some minor scientific issues:

1. Line 102: It seems appropriate to make a reference to Van Hellemond et al, PNAS 1998, who – still in the pro-genomics era, when studying acetate production in trypanosomatids – made the surprising finding of the ASCT/SCS cycle in trypanosomatids (although not in BSF T. brucei, but in procyclic T. brucei, Leishmania and Phytomonas). Until then (and still often), these enzymes were considered typical for anaerobic organisms.

2. Lines 133-134: I doubt if CMM better represents the in vivo situation than HMI medium. It may not be true with respect to amino acids. Probably it would be better to write 'which, with its 10 mM glucose, represents better – although still well above – the extracellular glucose concentration in the mammalian host’ (i.e., about 5 mM in human blood; 2-4 mM in ruminants). However, how relevant is it anyway, with glucose transport known to control the glycolytic flux (data from papers by Bakker and Haanstra), rendering it very unlikely that the intracellular glucose concentration is affected by changes in the external one at concentrations well above the Km of about 0.5-1 mM of the facilitated-diffusion transporter involved?

3. Lines 391-392: The information in this sentence is not accurately formulated, because it has been well known for years that metabolism of other substrates than glucose also contribute as carbon (i.e., for producing biomass) to T. brucei, such as amino acids and fatty acids. Glucose was considered the exclusive energy source, although in the presence of glycerol trypanosomes could be kept alive (by providing ATP) for some time without enabling them to proliferate. The research described in references 47 and 48 showed that, in the absence of glucose, glycerol can be used as both energy and carbon source. The novel finding of these references was that BSF can produce higher-molecular weight carbons from glycerol by gluconeogenesis, enabling cell proliferation.

4. Line 425: The suggestion that evasion of the immune response costs greater quantities of cytosolic ATP is unlikely. The major mechanism for evasion of the immune response is due to recycling the VSG coat, eliminating attached antibodies, driven by the trypanosome’s motility caused by flagellar beating. These processes also occur in vitro. Moreover, very recently published calculations show that this process (flagellar beating /motility /VSG recycling) involves only a very minor part of the BSF ATP budget (Nascimento et al., PLoS Pathog. 19(7): e1011522).

5. Lines 515-516: The (predominantly) cytosolic localization of alanine aminotransferase in BSF trypanosomes is not only ‘thought’ but has been clearly demonstrated by cell fractionation of trypanosomes, as reported in various publications. See for example Steiger et al., Eur. J. Biochem. 105, 1980, 163-175.

Textual and other minor issues:

1. Lines 4-5: Some authors are listed with their given name followed by their surname, while for other authors the surname is followed by the given name.

2. Line 126: Change ‘homology recombination’ to ‘homologous recombination’.

3. Line 136: Change 'was' to 'were'.

4. Line 176: At this first mentioning of the v5-tag (or in the legend of Fig. 3B), explain for non-informed readers the purpose of the tag (i.e., a peptide for immuno detection).

5. Lines 187 and 403: Change ‘mitochondria’ to ‘mitochondrion’.

6. Line 268-269: Change 'are' to 'is' and 'activity' to 'activities' (or change only 'were' in line 269 to 'was').

7. Line 302: 'downregulation' seems in this context a more appropriate word than 'impairment'.

8. Line 306: Alterations in TCA cycle metabolites were already mentioned above in lines 298-301.

9. Line 311: Add also ‘AAC DKO cells’ (they are shown in Fig. 8B, but this panel should be presented as 8C).

10. Line 318: Figure 8B should be 8C. Figure 8B (about AAC DKO cells) is not discussed in this section.

11. Line 347: 'intracellular'? Should it not be 'intramitochondrial'?

12. Line 429: Add 'in' to give: ‘when in in vivo or CMM-gly culture conditions'.

13. Line 431: 'Finally, we can conclude'? I suppose the authors meant something as: 'From all data together we can now conclude'.

14. Line 434: Change ‘occurs’ to 'was considered to occur'.

15. Lines 484-491: The text refers to Fig. 12A and 12C (and later – in line 549 - to 12D). No reference to panel B is made. I suggest adding such reference somewhere (because the panel is useful in the context of the other panels, and the journal would probably have all panels mentioned in the text).

16. Line 496: Correct to '10-times'.

17. Line 555: Add 'across the mitochondrial inner membrane' at the end of the sentence'.

18. Line 584: 'repressor under 10% T7 RNAP promoter'? The meaning of 10% is not clear to this reviewer.

19. Lines 588, 595, 606 and 619: The cassette/plasmid is not transfected but cells are transfected with the cassette/plasmid.

20. Line 594: Change 'inserted to' to 'inserted in’ (or into).

21. Line 625: Change 'was' to 'were'.

22. Line 632: Change 'with the sublethal concentration' to 'at the sublethal concentration.'

23. Lines 653 and 654: ‘coli’ with lower case c.

24. Line 721: Correct: °C (no superscript 0 but °) and labeled.

25. Line 785: Change AAC DKO cells to SCoAS DKO cells.

26. Lines 786 and 870: Delete 'the'.

27. Lines 805 and 882: Delete 'resistance' in front of ‘genes’ (it is redundant with the 2nd 'resistance' – linked to the antibiotic - later in the sentence).

28. Line 845: Add an ‘l’ to give ‘panel’.

29. Lines 849-850: Change 'levels bioluminescence' to 'bioluminescence levels'.

30. Line 918: (1) The panels of Figure 12 remain to be labeled A, B and C. (2) The panels are shown in the order (A) BSF427, (C) AAC DKO and (B) SCS DKO, not A, B and C.

31. Lines 945-946: The sentence is difficult to understand. Better (?): 'Data shown in the bars are derived from experiments of which representative graphs are shown in panel D.'

32. Figures 3D and F: Correct ‘luminiscence’ to ‘luminescence’.

Reviewer #2: Too many figures. Some proteomics and metabolomics data, like Fig. 4A & B, and 7A & B, could be presented in the supplementary material. As stated by the authors these analyses couldn’t detect any major metabolic changes.

Fig. 6E, SCS activity appears reduced in AAC DKO cells, although it was considered by the authors as non-significant. What is the basis for this consideration? How this reduction could be explained?

Discussion is too long, which needs to be shortened. Authors could focus on the novelty of their finding as discussed in the beginning of this section. It is also necessary to mention that studies were performed using the monomorphic BF. There are possibilities that DKO of AAC and SCS may generate slightly altered phenotypes than that observed here.

**********

PLOS authors have the option to publish the peer review history of their article (what does this mean?). If published, this will include your full peer review and any attached files.

If you choose “no”, your identity will remain anonymous but your review may still be made public.

**Do you want your identity to be public for this peer review?** For information about this choice, including consent withdrawal, please see our Privacy Policy.

Reviewer #1: **Yes: **Paul Michels

Reviewer #2: No

Figure Files:

While revising your submission, please upload your figure files to the Preflight Analysis and Conversion Engine (PACE) digital diagnostic tool, https://pacev2.apexcovantage.com. PACE helps ensure that figures meet PLOS requirements. To use PACE, you must first register as a user. Then, login and navigate to the UPLOAD tab, where you will find detailed instructions on how to use the tool. If you encounter any issues or have any questions when using PACE, please email us at figures@plos.org.

Data Requirements:

Please note that, as a condition of publication, PLOS' data policy requires that you make available all data used to draw the conclusions outlined in your manuscript. Data must be deposited in an appropriate repository, included within the body of the manuscript, or uploaded as supporting information. This includes all numerical values that were used to generate graphs, histograms etc.. For an example see here: http://www.plosbiology.org/article/info%3Adoi%2F10.1371%2Fjournal.pbio.1001908#s5.

Reproducibility:

To enhance the reproducibility of your results, we recommend that you deposit your laboratory protocols in protocols.io, where a protocol can be assigned its own identifier (DOI) such that it can be cited independently in the future. Additionally, PLOS ONE offers an option to publish peer-reviewed clinical study protocols. Read more information on sharing protocols at https://plos.org/protocols?utm_medium=editorial-email&utm_source=authorletters&utm_campaign=protocols

References:

Please review your reference list to ensure that it is complete and correct. If you have cited papers that have been retracted, please include the rationale for doing so in the manuscript text, or remove these references and replace them with relevant current references. Any changes to the reference list should be mentioned in the rebuttal letter that accompanies your revised manuscript. If you need to cite a retracted article, indicate the article’s retracted status in the References list and also include a citation and full reference for the retraction notice.

# Author comment: Author response to Decision Letter 0

**DOI:** 10.1371/journal.ppat.1011699.r002

15 Sep 2023

Attachment Submitted filename: Response to the reviewers_Taleva et al. FIN.docx Click here for additional data file.

# Editor report: Decision Letter 1

**DOI:** 10.1371/journal.ppat.1011699.r003

**Authors:** Laurie Read, James J Collins III

20 Sep 2023

Dear Alena,

We are pleased to inform you that your manuscript 'Mitochondrion of the*Trypanosoma brucei* long slender bloodstream form is capable of ATP production by substrate-level phoshorylation' has been provisionally accepted for publication in PLOS Pathogens.

Before your manuscript can be formally accepted you will need to complete some formatting changes, which you will receive in a follow up email. A member of our team will be in touch with a set of requests.

Please note that your manuscript will not be scheduled for publication until you have made the required changes, so a swift response is appreciated.

IMPORTANT: The editorial review process is now complete. PLOS will only permit corrections to spelling, formatting or significant scientific errors from this point onwards. Requests for major changes, or any which affect the scientific understanding of your work, will cause delays to the publication date of your manuscript.

Should you, your institution's press office or the journal office choose to press release your paper, you will automatically be opted out of early publication. We ask that you notify us now if you or your institution is planning to press release the article. All press must be co-ordinated with PLOS.

Thank you again for supporting Open Access publishing; we are looking forward to publishing your work in PLOS Pathogens.

Best regards,

Laurie Read

Pearls Editor

PLOS Pathogens

James Collins III

Section Editor

PLOS Pathogens

Kasturi Haldar

Editor-in-Chief

PLOS Pathogens

​orcid.org/0000-0001-5065-158X

Michael Malim

Editor-in-Chief

PLOS Pathogens

orcid.org/0000-0002-7699-2064

***********************************************************

Reviewer Comments (if any, and for reference):

# Editor report: Acceptance letter

**DOI:** 10.1371/journal.ppat.1011699.r004

**Authors:** Laurie Read, James J Collins III

3 Oct 2023

Dear Dr. Zíková,

We are delighted to inform you that your manuscript, "Mitochondrion of the*Trypanosoma brucei* long slender bloodstream form is capable of ATP production by substrate-level phosphorylation," has been formally accepted for publication in PLOS Pathogens.

We have now passed your article onto the PLOS Production Department who will complete the rest of the pre-publication process. All authors will receive a confirmation email upon publication.

The corresponding author will soon be receiving a typeset proof for review, to ensure errors have not been introduced during production. Please review the PDF proof of your manuscript carefully, as this is the last chance to correct any scientific or type-setting errors. Please note that major changes, or those which affect the scientific understanding of the work, will likely cause delays to the publication date of your manuscript. Note: Proofs for Front Matter articles (Pearls, Reviews, Opinions, etc...) are generated on a different schedule and may not be made available as quickly.

Soon after your final files are uploaded, the early version of your manuscript, if you opted to have an early version of your article, will be published online. The date of the early version will be your article's publication date. The final article will be published to the same URL, and all versions of the paper will be accessible to readers.

Thank you again for supporting open-access publishing; we are looking forward to publishing your work in PLOS Pathogens. 

Best regards,

Kasturi Haldar

Editor-in-Chief

PLOS Pathogens

​orcid.org/0000-0001-5065-158X

Michael Malim

Editor-in-Chief

PLOS Pathogens

orcid.org/0000-0002-7699-2064
